# Transcriptional Reorganization of *Drosophila* Motor Neurons and Their Muscular Junctions toward a Neuroendocrine Phenotype by the bHLH Protein Dimmed

**DOI:** 10.3389/fnmol.2017.00260

**Published:** 2017-08-14

**Authors:** Jiangnan Luo, Yiting Liu, Dick R. Nässel

**Affiliations:** Department of Zoology, Stockholm University Stockholm, Sweden

**Keywords:** transcription factor, neuromuscular junction, synapses, glutamate signaling, neuroendocrine cell

## Abstract

Neuroendocrine cells store and secrete bulk amounts of neuropeptides, and display morphological and molecular characteristics distinct from neurons signaling with classical neurotransmitters. In *Drosophila* the transcription factor Dimmed (Dimm), is a prime organizer of neuroendocrine capacity in a majority of the peptidergic neurons. These neurons display large cell bodies and extensive axon terminations that commonly do not form regular synapses. We ask which molecular compartments of a neuron are affected by Dimm to generate these morphological features. Thus, we ectopically expressed Dimm in glutamatergic, Dimm-negative, motor neurons and analyzed their characteristics in the central nervous system and the neuromuscular junction. Ectopic Dimm results in motor neurons with enlarged cell bodies, diminished dendrites, larger axon terminations and boutons, as well as reduced expression of synaptic proteins both pre and post-synaptically. Furthermore, the neurons display diminished vesicular glutamate transporter, and signaling components known to sustain interactions between the developing axon termination and muscle, such as wingless and frizzled are down regulated. Ectopic co-expression of *Dimm* and the insulin receptor augments most of the above effects on the motor neurons. In summary, ectopic *Dimm* expression alters the glutamatergic motor neuron phenotype toward a neuroendocrine one, both pre- and post-synaptically. Thus, Dimm is a key organizer of both secretory capacity and morphological features characteristic of neuroendocrine cells, and this transcription factor affects also post-synaptic proteins.

## Introduction

Neurons display a large variety of shapes, complexity of connectivity and molecular characteristics. For instance, motor neurons are characterized by a high precision in synaptic connections to specific muscles, possess extensive dendritic arborizations with precise somatotopic arrangements, and utilize small molecule neurotransmitters such as acetylcholine or glutamate (Budnik, 1996; Keshishian et al., 1996; Collins and DiAntonio, 2007; Menon et al., 2013; Darabid et al., 2014). In contrast, neuroendocrine cells are commonly peptidergic and do not form specific synaptic connections with their targets, but their axons terminate in diffuse areas for paracrine or hormonal release (Park et al., 2008; Nässel, 2009; Hamanaka et al., 2010; Nässel and Winther, 2010). Thus, we can distinguish efferent neurons of two main types, motor neurons and neuroendocrine cells with peripheral axon terminations. Although both may target muscle fibers, they display substantial molecular and morphological differences both centrally and peripherally. We ask here how the differentiation of these distinctive neuron types is regulated?

It has been established in *Drosophila* that the differentiation of upscaled neuroendocrine capacity in neurons is orchestrated by the bHLH transcription factor Dimmed (Dimm) (Hewes et al., 2003; Hamanaka et al., 2010; Park et al., 2011). Dimm confers a secretory phenotype to neuroendocrine cells that ensures increased capacity for neuropeptide production, storage, and release. This includes large cell bodies with well-developed endoplasmic reticulum and Golgi, enlarged storage capacity for neurosecretory vesicles associated with an upregulation of a host of specific proteins in the neuron (Hewes et al., 2003; Hamanaka et al., 2010; Hadzic et al., 2015). One intriguing finding was that ectopic Dimm suppresses expression of small molecule neurotransmitters, thereby seemingly promoting a peptidergic secretory phenotype (Hamanaka et al., 2010). We ask here whether Dimm also regulates changes in morphology of the neurons and their synaptic structures to further complete the neuroendocrine phenotype. To address this question we ectopically expressed Dimm in glutamatergic motor neurons that innervate specific body wall muscles in larval *Drosophila.* Thus, we investigated whether Dimm expression is sufficient to orchestrate a transformation of the motor neuron to a neuroendocrine cell, not only in terms of secretory capacity, but also with respect to morphology, synaptic structures, and other molecular characteristics.

In *Drosophila* motor neurons of the embryo and larvae are well-characterized in terms of lineages, central arborizations and muscle innervation patterns (Keshishian et al., 1996; Landgraf et al., 1997, 2003; Landgraf and Thor, 2006). There are relatively few motor neurons, and they are individually identifiable, have specific target muscle and are iterated in several segments of the organism. Thus, *Drosophila* motor neurons are excellent for analysis of developmental plasticity of synapse formation (Budnik, 1996; Keshishian et al., 1996; Collins and DiAntonio, 2007; Frank et al., 2013; Menon et al., 2013). Furthermore, the pre and post-synaptic features of the motor neuron terminations in muscles are characterized in detail, and the *Drosophila* neuromuscular junction (NMJ) therefore serves as an excellent genetic model for the development and plasticity in glutamatergic synapses in general (Budnik, 1996; Marrus et al., 2004; Rasse et al., 2005; Collins and DiAntonio, 2007; Menon et al., 2013). This well-established model NMJ is therefore excellent for our analysis of Dimm-induced transcriptional changes of neuron characteristics.

Our study shows that *Dimm* mis-expression is sufficient to cause a loss of glutamatergic phenotype in motor neurons and that this transformation also changes presynaptic structures of the axon termination as well as post-synaptic ones in the target muscle. Thus, the neurons lose presynaptic vesicular glutamate transporter (vGluT), bruchpilot, synapsin, synaptotagmin and post-synaptic disks large and glutamate receptors GluRIIA and B, all characteristic of *Drosophila* motor neurons. Interactions between the axon termination and muscle cell appear disrupted resulting in loss of wingless signaling components and formation of presynaptic filopodia-like structures. Furthermore, the *Dimm*-misexpressing motor neurons display morphologies similar to peptidergic efferent neuroendocrine cells and start to express several proteins known to be transcriptional targets of Dimm. In summary, we show that transcriptional orchestration by Dimm is sufficient to trigger a transformation of the NMJ toward a neuroendocrine phenotype both at the molecular and the morphological levels.

## Materials and Methods

### Fly Strains and Husbandry

All parental flies and larvae were reared at 25°C on a standard yeast, corn meal, agar medium, under 12:12 h light:dark conditions. Flies with other original backgrounds were backcrossed into *w^1118^* background for four generations before experiments.

The following Gal4 lines were used:

w; *Lk-*Gal4 (II) (gift from P. Herrero, Madrid, Spain),
w; Sco/Cyo; *RRa*-mcd8-GFP (Fujioka et al., 2003) (from R. Baines, Manchester, United Kingdom),
w; *Ok6*-Gal4;UAS-*Myc^Ric^*/TM3Sb, and w; UAS-*mcd8-gfp*/Cyo; *D42*-Gal4 (from A. Ferrus, Madrid, Spain).

The following UAS lines were employed:

w; UAS-*vGluT*-RNAi (II) [(104324) from The Vienna *Drosophila* Resource Center (VDRC), Vienna, Austria].
yw; UAS-*vGluT*-RNAi (III) (27538), yw; UAS-*dInR* (II), yw;UAS-*mcd8-gfp* (III) [all from Bloomington *Drosophila* Stock Center (BDSC), Bloomington, IN, United States],
w; UAS-*PI3K* (from A. Ferrus),
yw; UAS-*dimm-Myc* (III) (from P. H. Taghert, St. Louis, MO, United States),
w; UAS-*InR*; UAS-*dimm-Myc* (a recombination made in this lab),
w; If/Cyo, Dfd-GMR-YFP; *RN2-FLP14B*, tub < CD2 < Gal4, UAS*-myr::mRFP1(2x)/*Tm6b, Sb, Dfd-GMR-YFP (from M. Landgraf, Cambridge, United Kingdom).

### Antisera and Immunocytochemistry

The central nervous system (CNS) of first and third instar larvae as well as first and third instar larval body wall muscles were dissected in 0.1 M sodium phosphate buffer (PB; pH 7.4) and fixed in ice-cold 4% paraformaldehyde (4% PFA) in 0.1 M PB for 2–4 h. For anti-GluRIIA staining, the tissues were fixed in ice-cold 100% methanol for 10 min and rehydrated with 70, 50, and 20% methanol each for 5 min. All tissues were rinsed with 0.1 M PB three times over 1 h and washed finally in 0.01 M PBS with 0.25% Triton-X (PBS-Tx) for 15 min before application of primary antisera. The primary antisera were diluted in 0.01 M PBS-Tx with 0.05% sodium azide. Incubation with primary antiserum for whole tissues was performed for 24–48 h at 4°C with gentle agitation. For anti-Evi-N, anti-GluRIIB and anti-Wg staining, third instar larval body wall muscles were fixed in Bouin’s fixation for 20 min and washed intensively with 0.01 M PBS after fixation. All tissues were rinsed thoroughly with PBS-Tx, followed by application of secondary antibody overnight and thorough wash in 0.01 M PBS and then mounted in 80% glycerol in 0.01 M PBS.

For embryo analysis eggs were collected on food plates with apple juice at 25°C. After washing, embryos were dechorionated with 3% sodium hypochlorite for 2–3 min and blotted on paper to remove excess liquid. Embryos were fixed for 20 min with a 1:1 mixture of heptane and fresh 4% formaldehyde and devitellinized with ice-cold methanol for 30 s to 1 min. For immunostaining, embryos were rehydrated in 50% methanol for 5 min and washed 3 min × 5 min in PBST and then 4 min × 20 min in PBST with 0.5% NGS (normal goat serum). Incubation of primary antiserum was performed over night at 4°C with gentle agitation. Embryos were washed 3 min × 5 min and then 4 min × 20 min in PBST, and finally 30 min in PBS followed by application of secondary antiserum overnight at 4°C. For mounting, embryos were dehydrated 10 min each in 50, 70, 80, and 2 × 99% ethanol. Then embryos were clarified by replacing ethanol with methyl salicylate and settled for 30 min. Finally embryos were mounted in newly exchanged methyl salicylate.

The following primary antisera were used: guinea pig anti-Dimm (1:2000) [(Allan et al., 2005) from P. Taghert, St. Louis, MO, United States], rabbit anti-vGluT (1:1000) (from H. Aberle, Münster, Germany) (Mahr and Aberle, 2006), rabbit anti-horseradish peroxidase (1:500) (anti-HRP, # 323-005-021, Jackson ImmunoResearch), rabbit anti-proctolin (1:1000) (Taylor et al., 2004), mouse anti-synapsin (3C11) (1:100) mouse anti-nc82 (1:50), mouse anti-GluRIIA (8B4D2) (1:100), mouse anti-synaptotagmin (3H2 2D7) (1:10) from Developmental Studies Hybridoma Bank, Iowa, guinea pig anti-maelstrom (1:500) (Pek et al., 2009) from T. Kai, Univ. Singapore, guinea pig anti-CAT-4 (1:2000) (Park et al., 2011) and anti-PHM (1:750) (Jiang et al., 2000) both from P. Taghert, rabbit anti-GluRIIB (1:2500) from A. DiAntonio, St. Louis, MO, United States rabbit anti-Wg (1:500), rabbit anti-dfrizzled 2 (anti-DFz2C; 1:500), rabbit anti-Evi-Nex (1:100) all three from V. Budnik and J. Ashley, Worcester, MA, United States (Korkut et al., 2009), mouse monoclonal anti-disks large (Dlg; 1:2000) from DSHB (Parnas et al., 2001) and mouse monoclonal antibody to cysteine string protein (CSP) (Zinsmaier et al., 1990) was a gift from Dr. Eric Buchner (Würzburg, Germany).

To detect GFP we used rabbit, mouse and chicken anti-GFP (1:1000) (Invitrogen, Carlsbad, CA, United States), rat anti-GFP (1:1000) (Abcam, Cambridge, United Kingdom), and rabbit anti-RFP (1:5000) (Invitrogen). For detection of primary antisera the following secondary antisera were used: goat anti-rabbit Alexa 488, goat anti-mouse Alexa 488, goat anti-rat Alexa 488, goat anti-chicken Alexa 488, goat anti-rabbit Alexa 546, goat anti-mouse Alexa 546, goat anti-mouse Alexa 649, cy5 goat anti-rabbit (all from Invitrogen), Cy3-tagged goat anti-rat antiserum and Cy3-tagged goat anti-guinea pig antiserum (Jackson ImmunoResearch, West Grove, PA, United States) were all used at a dilution of 1:1000.

Rhodamine-phalloidin (Invitrogen) was employed at a dilution of 1:500 to stain muscle. DNA was visualized with 4′,6-Diamidine-2-phenylindole (DAPI; Sigma) at 1:2000.

### Image Analysis

Specimens were imaged with a Zeiss LSM 780 confocal microscope (Jena, Germany) using 20x, 40x, or 63x oil immersion objectives. Confocal images were processed with Zeiss LSM software for either projection of z-stacks or single optical sections. Images were in some cases edited for contrast and brightness in Adobe Photoshop CS3 Extended version 10.0.

For quantification of immunofluorescence confocal images of neurons from different genotypes were obtained with identical laser intensity and scan settings. Immunofluorescence intensity in both cell bodies and tissue background was quantified in a set of regions of interest using Image J 1.40 from NIH, Bethesda, MD, United States^1^ as described in Luo et al. (2013) and Liu et al. (2016). Mean fluorescence of projections of each cell body and image background were measured and the final immunofluorescence intensity in cell bodies was determined by subtracting the intensity of the tissue background. More specifically, for determining the background fluorescence intensity, 10 areas widely distributed on the muscle 1 (M1), but not on the aCC axon terminals, were randomly selected, and their mean fluorescent intensities were quantified using Image J. The average fluorescent intensity was calculated and used as background fluorescence. Total fluorescence of cell bodies was calculated as mean fluorescence multiplied with cell size in some cases. For each genotype neurons in 5–15 animals were measured.

For size determination, the outline of each cell body was extracted and its area determined using Image J. For each genotype neurons in 5–15 larvae from three independent crosses were measured. For determination of axon and arborization size in larval body wall muscle, staining of NMJ in M1-2 from the third segment was imaged and then quantified by outlining of each axon using Image J. For each genotype neurons in at least five body walls were measured. Boutons from mCD8-GFP-labeled axon terminals from the third segment were counted, and the area of each bouton was manually outlined and quantified using Image J. The average bouton size from individual axon terminals was calculated and the data set from 5 to 15 larvae of each genotype was plotted. The data were analyzed in Prism GraphPad 6.0, with Student’s *t*-test, one way ANOVA followed by Dunnett’s multiple comparison or two way ANOVA followed by Tukey’s multiple comparison. All data are presented as mean values ± SEM.

## Results

### Characteristics of Glutamatergic Motor Neurons and Efferent Peptidergic Neuroendocrine Cells

Since we are interested in transcriptional changes that may shift a glutamatergic motor neuron toward a phenotype typical of efferent peptidergic neurons (neuroendocrine cells), we first highlight the characteristics of these two types of neurons.

**FIGURE 1:**
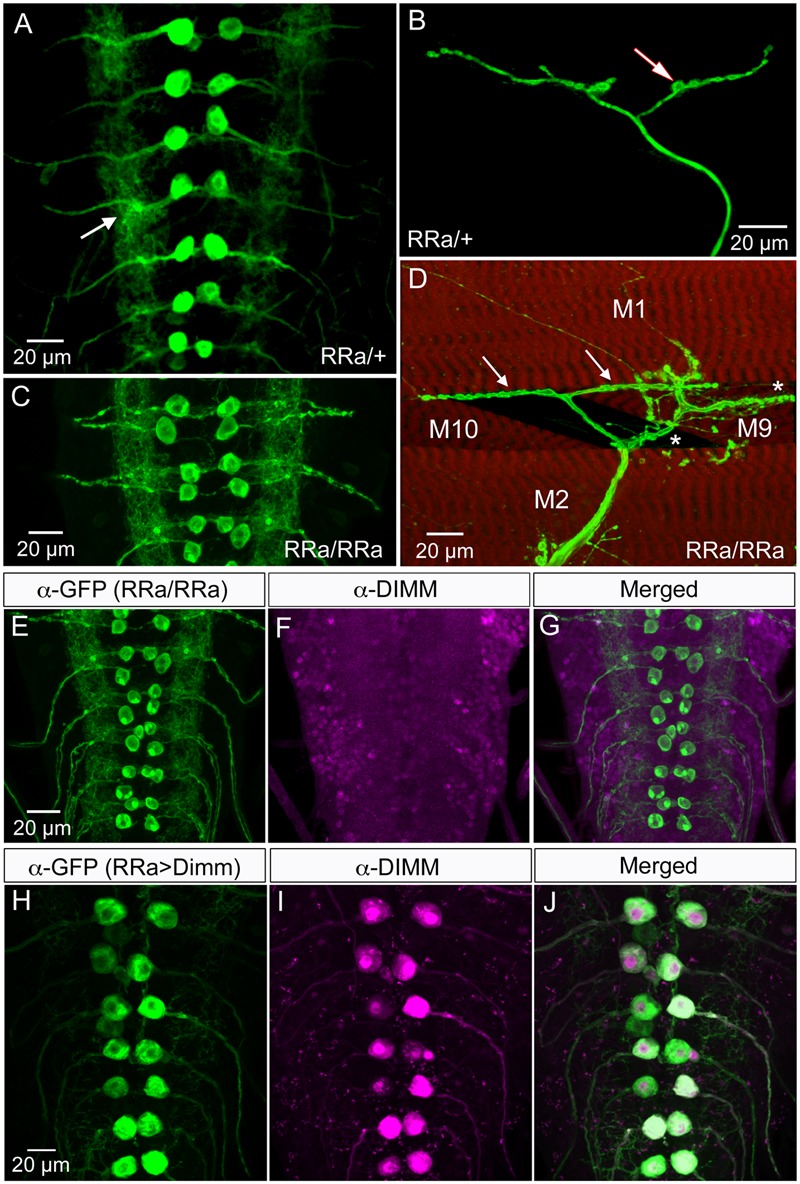
The larval aCC motor neurons are Dimm negative and terminate on abdominal muscle 1. **(A)** The RRa-Gal4 driver recombined with GFP displays the aCC motor neurons in the ventral nerve cord (one pair in each neuromere) of third instar larva. The aCC dendrites are seen laterally (arrow). **(B)** The aCC axon termination on muscle 1. One bouton is indicated by arrow. **(C)** Using a homozygous RRa-Gal4 driver both the anterior aCC and the posterior RP2 neurons display GFP in each segment. **(D)** With the homozygous RRa-Gal4 driver axon terminations are seen on muscles 1, 2, 9, and 10. The termination from aCC on muscle 1 is indicated by arrows. **(E–G)** The aCC and RP2 neurons are DIMM negative as seen with double labeling with anti-GFP and anti-DIMM. Scale bar in **(A)** is also for **(F,G)**. **(H–J)** Ectopic expression of Dimm in aCC neurons (RRa-Gal4 > Dimm) triggers growth of the cell bodies and strong expression of anti-DIMM, both in nuclei and cytoplasm. Scale bar in **(H)** is also for **(I,J)**.

Glutamatergic motor neurons in the larval ventral nerve cord have segmentally arranged cell bodies with relatively large diameters. Their dendrites are distinct with spatially well-defined wide arborizations, their axon terminations are restricted to specific muscles and their presynaptic boutons are large and numerous (Keshishian et al., 1996; Landgraf et al., 1997; Sanchez-Soriano et al., 2005; Landgraf and Thor, 2006; Kim et al., 2009). We primarily analyzed a specific motor neuron type in abdominal neuromeres, the aCC neurons, that innervate body wall muscle 1 (M1), which in late third instar larvae are labeled by the RRa-Gal4 driver (Fujioka et al., 2003; Lin et al., 2012) (**Figures 1A,B**). Importantly, the larval motor neurons, including the aCC neurons, do not express Dimm (Park et al., 2008) (see **Figures 1E–G**).

In contrast, the efferent peptidergic neurons commonly have poorly defined dendrites, and extensive thin axon terminations with small boutons (Budnik, 1996; Keshishian et al., 1996; Santos et al., 2007; Nässel and Winther, 2010). These terminations are not always muscle specific, and may also have additional targets. Here, as a reference, we show the segmental abdominal efferent neurons expressing the peptide leucokinin (LK) (see Supplementary Figure 1). These neurons designated ABLK neurons, specified by an Lk-Gal4 driver (de Haro et al., 2010), associate primarily with larval body wall muscle 8 (M8), but may also supply short branches to adjacent muscles (Cantera and Nässel, 1992; Luo et al., 2013). The axons of the ABLK terminations associated with M8 are thin with small boutons (Supplementary Figure 1).

It should be noted that although several types of larval motor neurons express the neuropeptide proctolin (Anderson et al., 1988; Taylor et al., 2004), the aCC neurons do not (Supplementary Figure 2). However, the adjacent large motor neurons RP2 do produce proctolin, although with some variability (Supplementary Figure 2). Proctolin is a non-amidated peptide that does not seem to require Dimm or PHM for its expression (Park et al., 2008). In support of this we found here that both aCC and RP2 neurons are indeed Dimm negative (**Figures 1E–G**).

### Ectopic Expression of *Dimm* in Motor Neurons Affects the Size of Cell Bodies, Dendrites, and Axon Terminations

To introduce Dimm into motor neurons, we used the RRa-Gal4 driver (Fujioka et al., 2003) fused with GFP to selectively drive expression of target genes in the segmental aCC motor neurons. In third instar larvae the aCC motor neurons innervate body wall muscle 1 (M1) in the corresponding segment and have large cell bodies anteriorly in each abdominal hemi-segment of the ventral nerve cord (**Figures 1A,B**). In larvae homozygous for the RRa-Gal4 the GFP expression is additionally seen in RP2 neurons (**Figure 1C**). RP2 innervates muscles 2, 9, and 10 (**Figure 1D**). The aCC neurons form a restricted axon termination on M1 with a small number of relatively large boutons (**Figure 1B**). Furthermore, the aCC neurons have distinct dendrites along the midline of the neuropil (in both hemispheres) of the nerve cord (**Figure 1A**). We confirmed that the aCC (and RP2) neurons are glutamatergic by immunolabeling with antiserum to a vGluT (see Supplementary Figure 4A).

**FIGURE 2:**
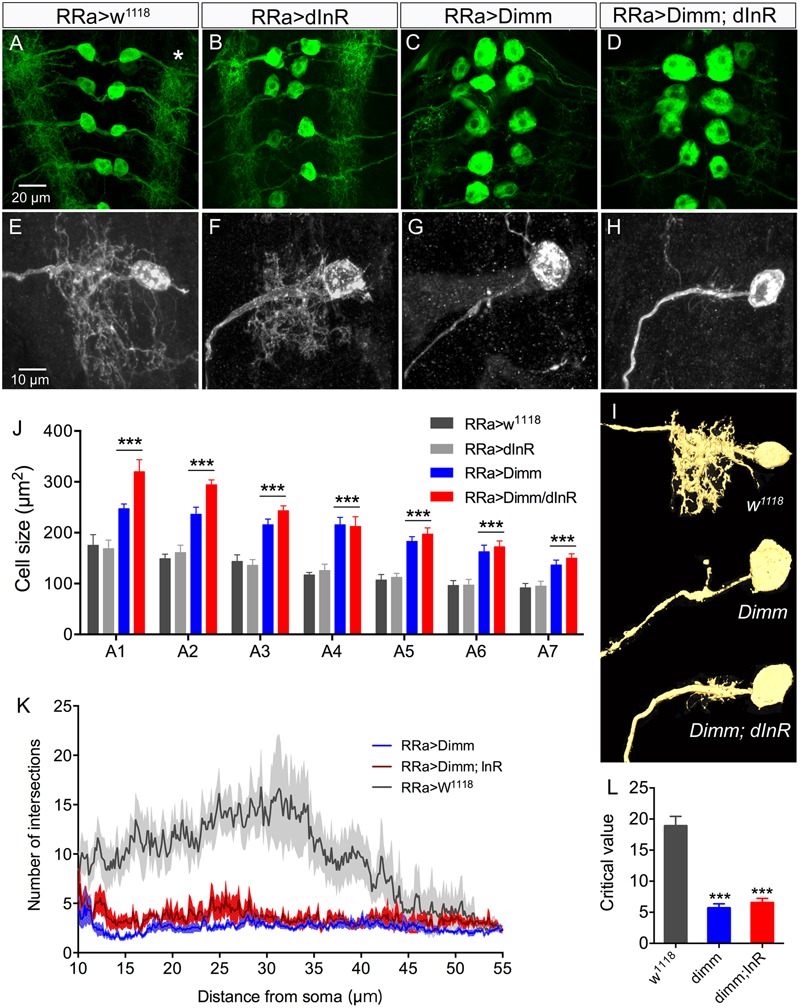
Ectopic expression of Dimm induces growth of aCC cell bodies and loss of growth of their dendrites. **(A–D)** The size of the aCC cell bodies is increased by ectopic expression of Dimm and Dimm combined with insulin receptor (Dimm; dInR), but not by dInR alone (visualized by RRa; GFP and anti-GFP). **(E–H)** The dendrites of the aCC neurons in abdominal segment A4 are reduced after expression of Dimm **(G)** and Dimm; dInR **(H)**. These images are obtained by utilizing an RN2-FLP construct and mild heat shock late in the embryo to generate small, random subpopulations of aCC/RP2 neurons for driving Dimm and Dimm+dInR expression (visualized by red fluorescent protein). **(I)** Amira renderings of representative aCC neurons from A4 showing the ipsilateral dendrites. Note that in A4 the additional effect of dInR expression is negligible. **(J)** Quantification of cell body sizes after ectopic expression of Dimm or Dimm+dInR compared to controls and dInR alone. Abdominal segments A1–A7 were analyzed; both Dimm and Dimm+dInR expression induced significantly increased cell bodies in all segments compared to RR > w1118 and RRa > dInR crosses (^∗∗∗^*p* < 0.001, as assessed by two way ANOVA followed by Tukey’s multiple comparisons test). The additional effect of combined ectopic dInR and Dimm compared to ectopic dimm alone (blue to red bars) was only significant in A1 and A2 (*p* < 0.05 for A1 and *p* < 0.01 for A2 for A2 as assessed by unpaired *t*-test). Data are presented as means ± SEM; with 9–12 larvae from three biological replicates. **(K)** Sholl profiles of dendrite density, measured between 10 and 55 μm from the cell body (soma). Number of intersections for dendrite of aCC neurons after Dimm and Dimm+dInR expression were much fewer than control larvae. **(L)** The peak of maximum branch density was determined by critical value (Ristanovic and Milosevic, 2007), it was significantly reduced after Dimm and Dimm+dInR expression. Data are presented as means ± SEM; with 7–8 larvae from three biological replicates (^∗^*p* < 0.05, ^∗∗∗^*p* < 0.001, ns, not significant), as assessed by Mann–Whitney test after Shapiro–Wilk normality test.

*Dimm* mis-expression in aCC neurons was verified by detecting Dimm immunolabeling in the nuclei and cell bodies in the ganglion (**Figures 1H–J**). We found that the Dimm immunolabeling was further increased by simultaneously mis-expressing *Dimm* and the insulin receptor (*dInR*) in the aCC neurons (not shown). We had shown previously that expressing the dInR in *Dimm*-positive neurons leads, not only to increased Dimm immunolabeling, but it also increased the size of the cell body (Luo et al., 2013; Liu et al., 2016). *Dimm* mis-expression in aCC neurons leads to a drastic increase in the size of their cell bodies (**Figures 2A–C,J**). This growth effect was amplified by co-expression of *Dimm* and *dInR* in the neurons, but only in abdominal segments A1 and A2 the extra growth was significant (**Figures 2D,J**). Since the ectopic co-expression of dInR was found to enhance the effect of Dimm on cell body size we tested the effects of Dimm+dInR in subsequent experiments with other markers to determine whether also other phenotypes were exacerbated. We, however, did not express dInR alone in the Dimm negative aCCs (except in the experiment shown in **Figure 2**), since our previous work suggest that ectopic dInR excerts effects on growth only in the presence of Dimm (Luo et al., 2013; Liu et al., 2016). This is confirmed in **Figures 2B,F,J** where aCC cell bodies and dendrites are unaffected by ectopic dInR.

In addition to the enlarged cell bodies, the ectopic *Dimm* and *Dimm+dInR* expression resulted in drastically reduced dendritic branching in the aCC neurons (**Figures 2E–I**). For a detailed analysis we utilized an RN2-FLP construct and mild heat shock late in the embryo to generate small, random subpopulations of aCC/RP2 neurons for driving *Dimm* and *Dimm+dInR* expression. Dendritic arborizations of single motor neurons from the same abdominal segment were compared. Overexpression of *Dimm* and *Dimm+dInR* in these motor neurons resulted in enlarged cell bodies and a nearly complete loss of dendrites (**Figures 2E–H**), while *dInR* overexpression alone did not have any impact on cell body size, or the dendritic arborizations (**Figures 2B,F**). We show 3-D rendering of dendrites in **Figure 2I** and quantifications of density of dendrite branching in **Figures 2K,L**.

**FIGURE 3:**
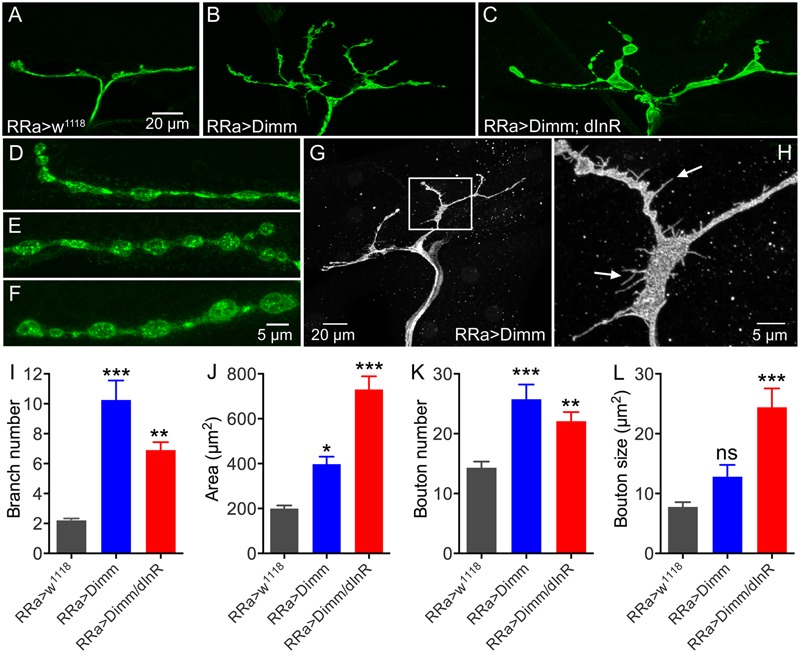
The axon terminations of the aCC neurons enlarge after ectopic expression of Dimm and Dimm/dInR. **(A–C)** Axon terminations of aCC neurons on muscle 1 enlarge and display more branches after Dimm expression. This is accentuated by coexpression of the dInR **(C)**. **(D–F)** Details of boutons of axon terminations (**D**, RRa > w1118; **E**, RRa > Dimm; **F**, RRa > Dimm; dInR). **(G,H)** After ectopic expression of Dimm the axon terminations commonly display filopodia-like protrusions, detail of inset in **(G)** is shown in **(H)** at higher magnification (arrows indicate filopodia-like protrusions). **(I–L)** Quantification of branch number **(I)**, branching area **(J)**, bouton number **(K)** and bouton size of aCC axon terminations in control, Dimm and Dimm+dInR expressing neurons. Data are presented as means ± SEM; with 8–11 larvae from three biological replicates (^∗^*p* < 0.05, ^∗∗^*p* < 0.01, ^∗∗∗^*p* < 0.001, ns, not significant, as assessed by one way ANOVA followed by Tukey’s multiple comparisons test).

Furthermore, ectopic *Dimm* and *Dimm+dInR* expression induced increases in the size and number of branches of axon terminations on M1 (**Figures 3A–C**). Also the boutons in the terminations are larger after *Dimm* mis-expression (**Figures 3D–F**). We found that the enlarged boutons frequently display thin filopodia-like protrusions over the muscle surface (**Figures 3G,H**) that were not seen in controls. Quantifications of changes in axon terminations are shown in **Figures 3I–L**. Expression of the *dInR* without co-expressing *Dimm* in aCC neurons has no effect on the boutons or axon branching of the terminations on M1 (not shown).

To examine at what time point ectopic Dimm induces altered development we analyzed embryonic aCC/RP2 neurons at stages 14–16. Earlier studies have shown that axon outgrowth from aCC neurons starts at about stage 13 and reaches the muscle at about stage 16, after which growth cones start exploring the muscle fiber (Keshishian et al., 1996; Yoshihara et al., 1997). We find that the RRa-Gal4 drives GFP in aCC/RP2 motor neurons at stage 16 and that RRa-Gal4 > Dimm induces growth of cell bodies at this stage (Supplementary Figures 3A–C). At this time point we detected GFP labeled axon terminations on the body wall muscles (Supplementary Figure 3D). Effects of *Dimm* expression in aCC and RP2 neurons were also visible in axon terminations in late first instar larvae: axon terminations were increased (Supplementary Figures 3E,F). Since the aCC neurons find the correct muscle (and area in muscle), we assume that Dimm-induced effects on axon terminals set in around stage 16, after muscles are reached and do not affect axonal pathfinding.

### Mis-expression of Dimm in Motor Neurons Diminishes Vesicular Glutamate Transporter (vGluT) and Presynaptic Proteins

**FIGURE 4:**
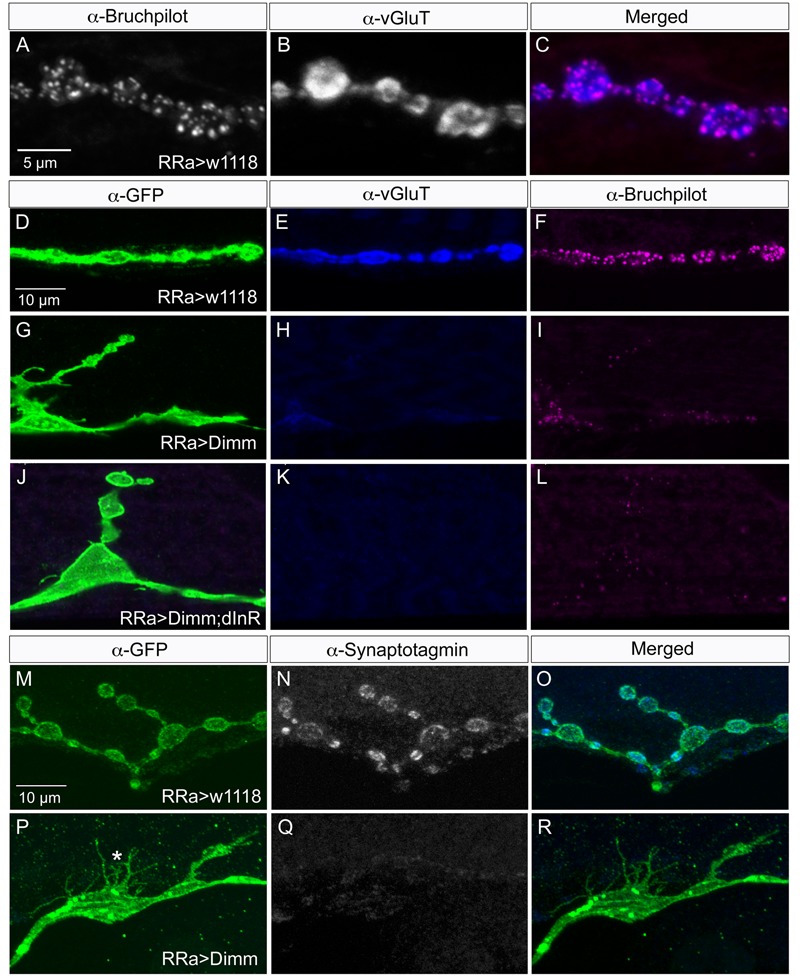
Synaptic proteins are affected by ectopic Dimm expression. Note that in this and the following figures the axon terminations are shown in Z-stacks of several optic sections and thus the entire volume of the axons is commonly shown (this also means that often adjacent axon terminations on nearby muscle fibers may be depicted). **(A)** In control larvae the active zone protein bruchpilot (Brp) identifies pre-synaptic sites in the aCC axon termination (visualized by nc82 antibody). **(B)** The vesicular glutamate transporter (vGluT) is detected presynaptically. **(C)** Merged images. **(D–L)** Ectopic expression of Dimm and Dimm+dInR strongly reduces immunolabeling for vGluT and Brp. Scale bar in **(D)** is also for **(E–L)**. **(M–R)** Ectopic expression of Dimm strongly reduces immunolabeling for synaptotagmin in pre-synaptic boutons. Scale bar in **(M)** is also for **(N,O)**. Data for effects of Dimm+dInR expression on synaptotagmin are shown in **Figure 6**.

**FIGURE 5:**
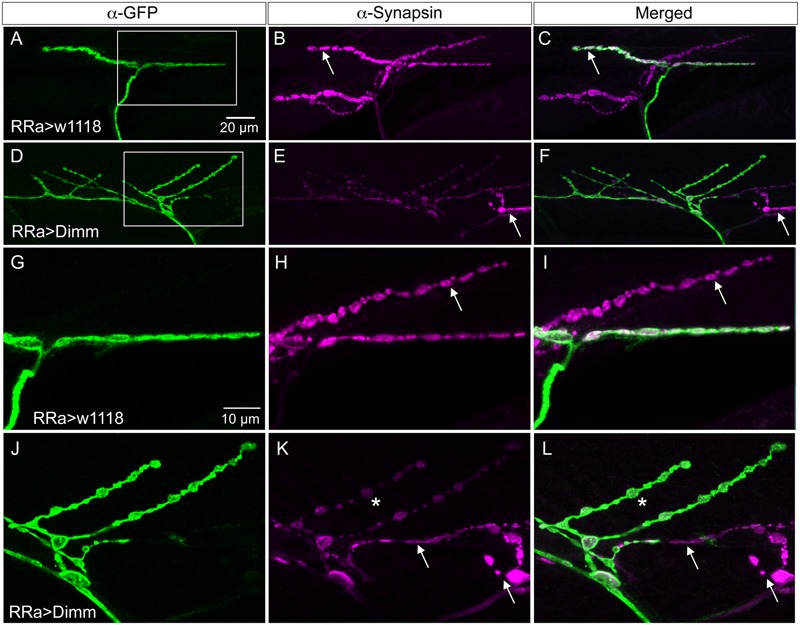
Synapsin expression is reduced by ectopic Dimm expression. **(A–C)** In control larvae synapsin immunolabeling is seen throughout the axon termination. Here an additional (GFP-negative) synapsin-expressing termination is seen on an adjacent muscle (arrow). Scale bar in **(A)** is also for **(B–F)**. **(D,E)** Ectopic Dimm expression reduces synapsin immunolabeling in the aCC neurons, but not in an adjacent neuron (arrow). **(G–L)** The same axon terminations are shown in higher magnification. Arrows indicate adjacent GFP-negative neurons and asterisks in **(K,L)** indicate branches of the aCC axon termination in which synapsin immunolabeling is reduced. Note that in **(I,L)** a third marker is added: anti-vesicular glutamate transporter (mostly obscured by the anti-synapsin). Scale bar in **(G)** is also for **(H–L)**. Data for effects of Dimm+dInR expression on synapsin are shown in **Figure 6**.

*Drosophila* motor neurons are glutamatergic (Jan and Jan, 1976; Johansen et al., 1989) and express a number of presynaptic proteins such as for instance synapsin, synaptotagmin, and bruchpilot (see Collins and DiAntonio, 2007; Menon et al., 2013). A vGluT is required for transporting the neurotransmitter into the synaptic vesicles and serves as a reliable marker for glutamatergic neurons (Daniels et al., 2004; Mahr and Aberle, 2006). We validated that the aCC motor neurons express vGluT, and the presynaptic proteins bruchpilot (Brp), synapsin, and synaptotagmin (see **Figures 4, 5**). As seen in Supplementary Figure 1 there is no vGluT labeling and Brp expression is very scarce in peptidergic axon terminations.

**FIGURE 6:**
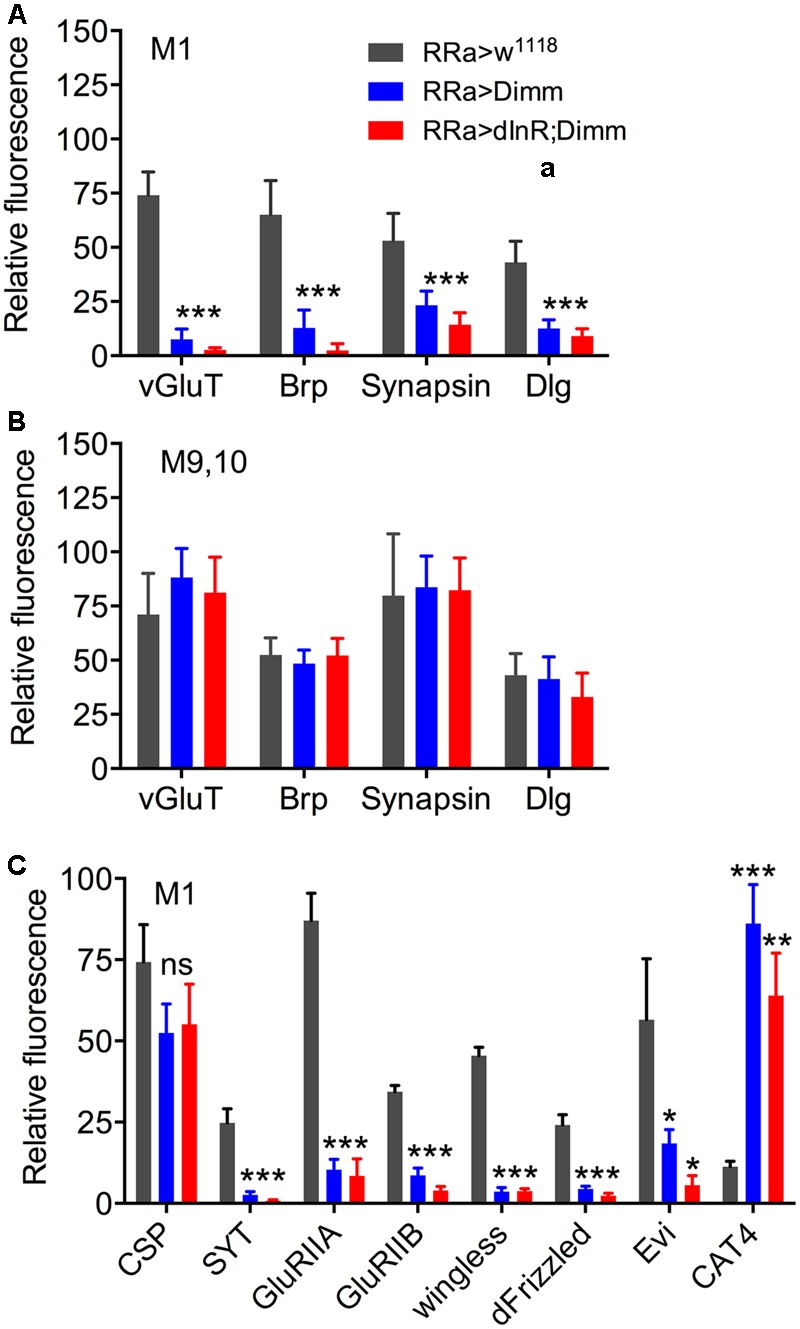
Quantification of Dimm-induced changes in synaptic markers in axon terminations. **(A)** Relative changes in immunolabeling levels in terminations of aCC neurons (muscle 1; M1) of vGluT, bruchpilot (Brp), synapsin, and disks large (Dlg). Both for ectopic Dimm and Dimm+dInR expression all the markers were significantly reduced. Data are presented as means ± SEM; with 7–9 larvae from three biological replicates (^∗^*p* < 0.05, ^∗∗^*p* < 0.01, ^∗∗∗^*p* < 0.001), as assessed by two way ANOVA test followed by Tukey’s multiple comparisons test. **(B)** As a control markers in axon terminations on muscle 9/10 (not innervated by aCC neurons) were recorded. No significant changes in maker expression were seen in these terminations. **(C)** Relative changes in immunolabeling levels in terminations of aCC neurons (muscle 1; M1) of cysteine string protein (CSP), synaptotagmin (SYT), glutamate receptor IIA (GluRIIA) and IIB (GluRIIB), wingless, frizzled, evenless interrupted (Evi) and the transporter CAT4. Dimm and Dimm+dInR expression induces no change in CSP, increases CAT4, and decreases the other markers. Data are presented as means ± SEM; with 8–10 larvae from three biological replicates (ns, not significant), as assessed by two way ANOVA test followed by Tukey’s multiple comparisons test.

Next we examined whether *Dimm* mis-expression affect the glutamatergic phenotype of the aCC neurons using the RRa-Gal4 driver. Targeted *Dimm* expression strongly reduced vGluT immunolabeling in the cell bodies (Supplementary Figures 4A,B) and axon terminations of aCC neurons (**Figures 4A–L**). Also the active zone protein Brp, as detected with the antiserum nc82, was drastically diminished (RRa > *Dimm*) or no longer detectable presynaptically (RRa > *Dimm; dInR*) (**Figures 4D–L**). Furthermore, we found that synaptotagmin immunolabeling was no longer visible (**Figures 4M–R**) and synapsin labeling drastically reduced (**Figure 5**). When driving *Dimm* expression combined with *dInR* in aCC neurons we observed the same phenotypes as above and here bruchpilot was consistently non-detectable (**Figures 4J–L**). Quantification of the decreases in immunolabeling is shown in **Figures 6A–C**. As internal control we measured the same markers in Dimm-negative boutons on the adjacent muscles 9 and 10 (**Figure 6B**). Thus, the *Dimm* mis-expression diminishes levels of several markers typical of glutamatergic neurons and dInR coexpression amplifies the effect.

We verified the growth of cell bodies and loss of vGluT immunolabeling using a broad motor neuron driver, OK6-Gal4, known to be expressed in most of the motor neurons and some interneurons (Sanyal, 2009). Both *Dimm* and *Dimm+dInR* mis-expression lead to diminished vGluT immunolabeling in the enlarged cell bodies of large midline motor neurons, and dInR alone produced a slight non-significant increased vGluT (Supplementary Figures 4C,D). Moreover, we noted that the effect of *Dimm* mis-expression with the OK6-driver lead to lethality where most offspring died as late third instar larvae. The few surviving larvae displayed diminished body size and resulted in small pupae, probably due to reduced food intake. Furthermore the dwarfed OK6 > *Dimm* larvae displayed abnormal crawling and a tail-up phenotype, indicating that muscle coordination was severely impaired.

Another, more specific, driver, D42-Gal4 (fused with mcd8-GFP) was used to drive *Dimm* and *Dimm+dInR* to confirm the vGluT and growth phenotype. D42 displays a broader expression pattern than the RRa-Gal4, but smaller than OK6. However, mis-expression of *Dimm* and *Dimm+dInR* using this strong driver led to lethality at late embryonic stage (data not shown).

### Mis-expression of Dimm in Motor Neurons Affects Post-synaptic Structures

Since we detected a loss of vGluT and several presynaptic markers in aCC motor neurons after *Dimm* mis-expression, we next tested whether this manipulation also affected post-synaptic structures in M1. The post-synaptic portion of the NMJ is characterized by a subsynaptic reticulum (SSR) and attached ionotropic glutamate receptors (iGluR) of a few types (Marrus et al., 2004; Collins and DiAntonio, 2007). To label the SSR we used an antiserum to disks large (DLG), a prototypic member of a family of membrane-associated guanylate kinase homologs (MAGUKs), known to be associated with the SSR (Lahey et al., 1994; Menon et al., 2013).

**FIGURE 7:**
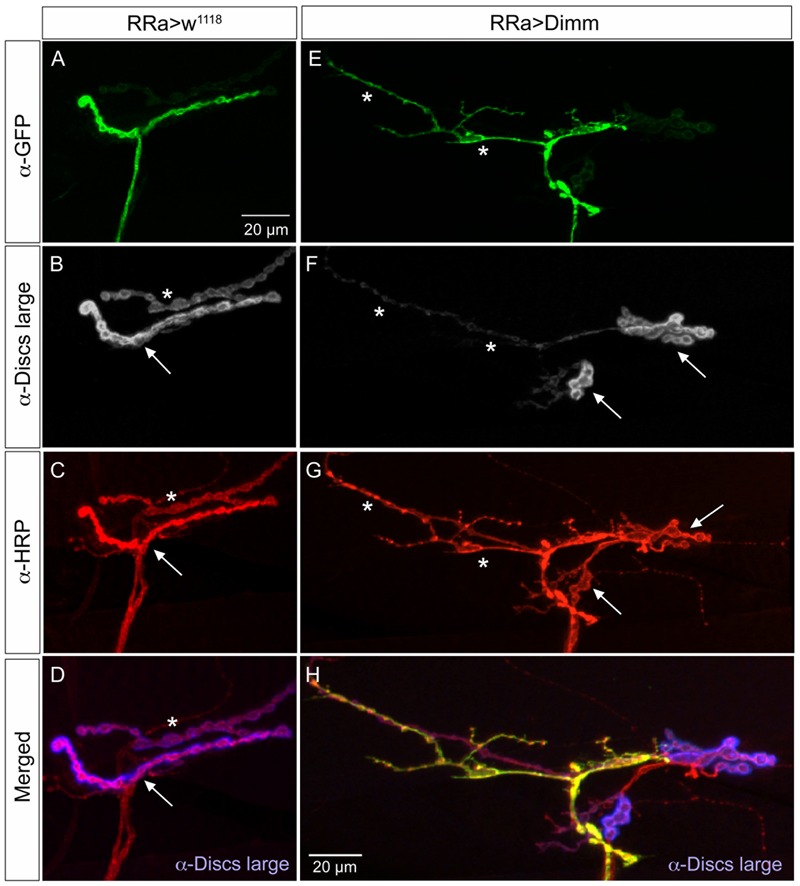
The expression of the post-synaptic marker disks large is reduced by ectopic Dimm expression. **(A–D)** The marker for the subsynaptic reticulum (SSR), disks large (Dlg) is prominent around the boutons of the control axon termination (of aCC neuron), indicated by arrow in **(B)**. Another termination is seen (asterisk – see also **C**). Scale bar in **(A)** is also for **(B–G)**. **(C)** Anti-HRP labels the membranes of the aCC neuron (arrow) and another one (asterisk) that is Dimm-negative. **(D)** Merged image. Here the Dlg is shown in blue. **(E–H)** Ectopic Dimm expression in aCC neurons (RRa > Dimm) eliminates Dlg immunolabeling from the GFP-positive aCC neurons (asterisks in **E–G**), but it remains in an adjacent neuron that can be seen after anti-HRP labeling (arrows) in **(G)**. **(H)** A triple labeling clearly shows that Dlg immunolabeling (blue) is only detectable in the adjacent neuron. Data for effects of Dimm+dInR expression are shown in **Figure 6**.

In the larvae with *Dimm* and *Dimm+dInR* mis-expression in aCC neurons DLG immunostaining was lost in M1 (**Figures 6A, 7**), whereas in adjacent muscles DLG labeling of post-synaptic sites of Dimm-negative motor neurons remained intact. This finding suggests that loss of presynaptic structures and presumably defect glutamate-loading of synaptic vesicles affects the post-synaptic SSR. Note that peptidergic axon terminations on abdominal muscle lack associated Dlg immunolabeling (Supplementary Figure 1E).

**FIGURE 8:**
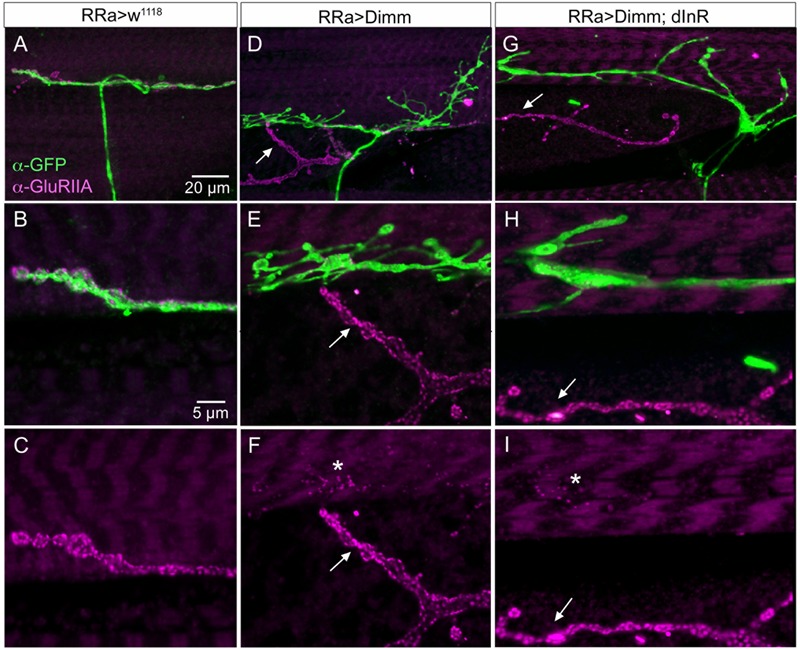
The expression of the post-synaptic glutamate receptor GluRIIA is strongly reduced by ectopic Dimm expression. **(A–C)** Post-synaptic to control aCC neurons GluRIIA immunolabeling is prominent. Scale bar in **(A)** is also for **(D,G)**; scale bar in **(B)** is also for **(E–I)**. **(D–F)** After Dimm expression in aCC neurons GluRIIA immunolabeling is strongly reduced (asterisk). In the same region an adjacent GFP-negative axon termination (arrow) displays strong GluRIIA immunolabeling. **(G–I)** Combined Dimm and dInR expression in aCC neuron also reduces the GluRIIA immunolabeling, whereas in an adjacent termination (arrow) the labeling is unaltered.

**FIGURE 9:**
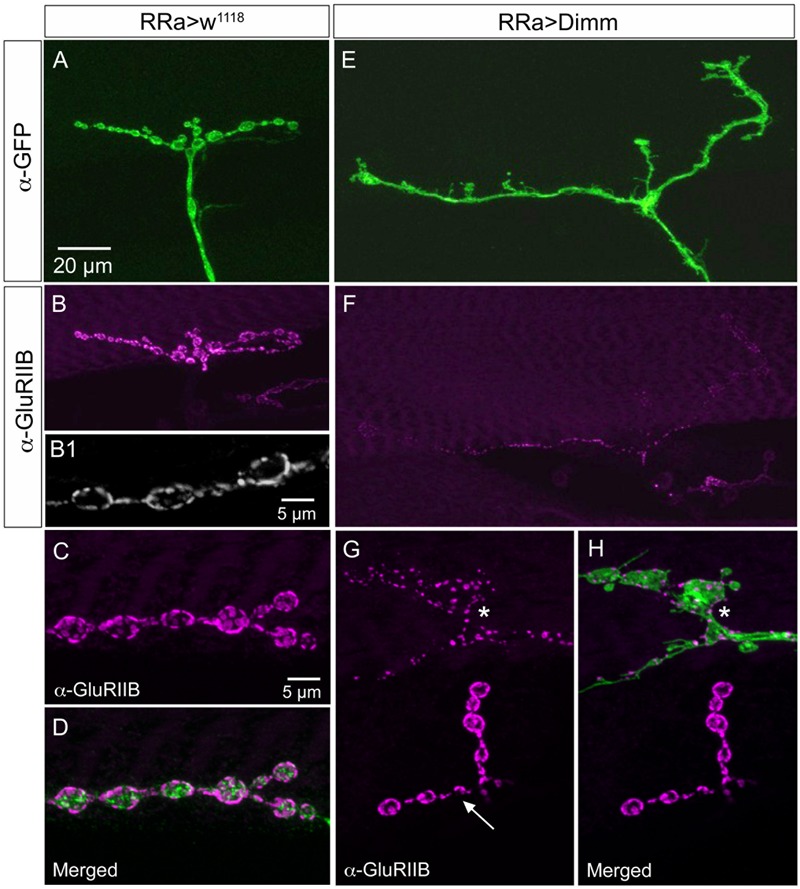
The expression of the post-synaptic glutamate receptor GluRIIB is reduced by ectopic Dimm expression. **(A–D)** GluRIIB immunolabeling is prominent post-synaptic to control aCC neurons. In **(B1,C,D)** enlarged portions of smaller Z-projections are shown (from other specimen). Scale bar in **(A)** is also for **(B,E,F)**; scale bar in **(C)** is also for **(D,G,H)**. **(E,F)** After Dimm expression in aCC neurons GluRIIB immunolabeling is strongly reduced. **(G,H)** After Dimm expression in different aCC neuron (asterisk) the GluRIIB immunolabeling is strongly reduced, whereas in an adjacent termination (arrow) the labeling is unaltered (projections of complete Z-stacks). Data for effects of Dimm+dInR expression are shown in **Figure 6**.

The glutamate-gated iGluRs in the *Drosophila* NMJ are tetrameric proteins composed of the obligatory subunits GluRIIC, D, E and either GluRIIA (type A receptor) or GluRIIB (type B) (Marrus et al., 2004; Qin et al., 2005; Collins and DiAntonio, 2007). We employed antisera to GluRIIA and GluRIIB (Marrus et al., 2004) to probe post-synaptic effects of the *Dimm* and *Dimm+dInR* mis-expression in aCC neurons and found that both manipulations very strongly diminished labeling for GluRIIA (**Figures 6C, 8**) and GluRIIB (**Figures 6C, 9** and Supplementary Figure 5). In adjacent muscles, including the M10 innervated by the RP2 neurons, the receptor immunolabeling was intact. Thus, the manipulation of the presynaptic structures in aCC neurons affected the SSR and expression of post-synaptic glutamate receptors just adjacent to the presynaptic structures.

### Wingless Signaling Is Affected by Dimm Expression in Motor Neurons

**FIGURE 10:**
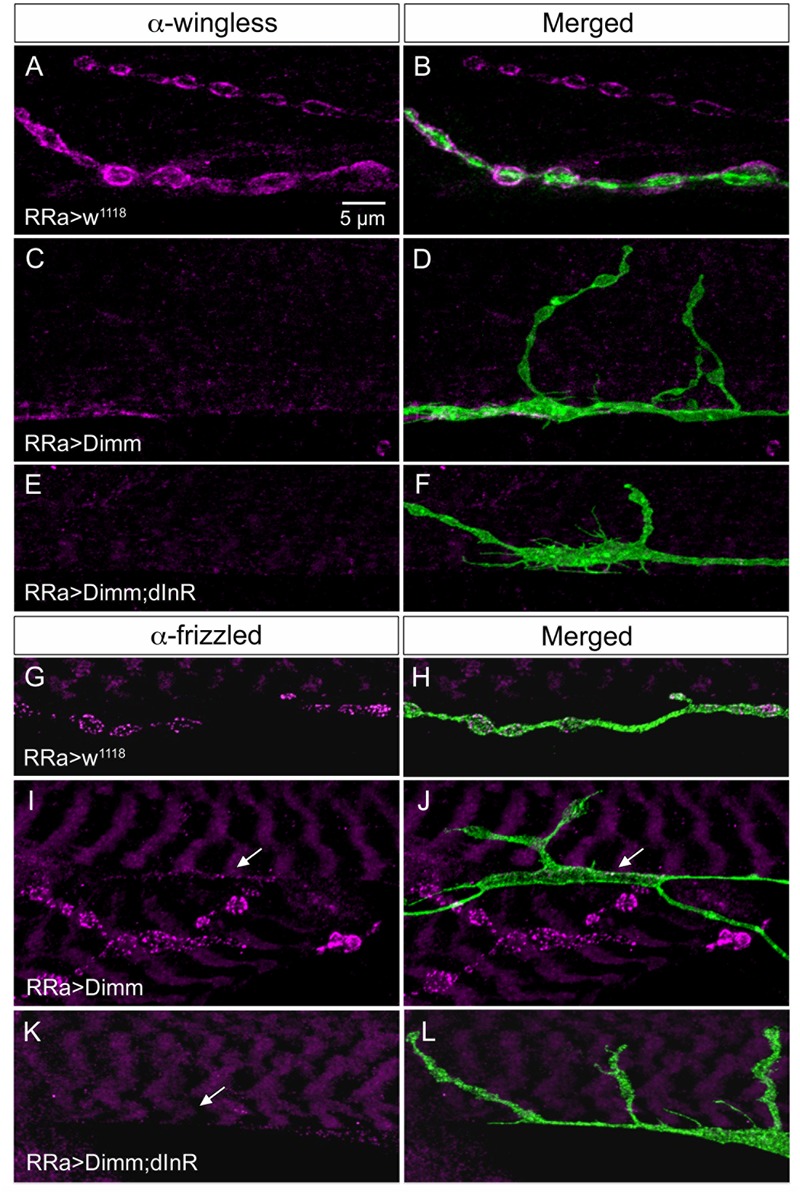
The expression of wingless and frizzled-2 is affected by ectopic Dimm in aCC neurons. **(A–F)** Antiserum to wingless (Wg) labels pre- and post-synaptic structures adjacent to boutons in control larvae **(A,B)**, but immunolabeling is nearly absent after Dimm **(C,D)** and Dimm; dInR **(E,F)** expression in aCC neurons. Scale bar in **(A)** is also for **(B–L)**. **(G–L)** Antiserum to frizzled-2 (dFz2) labels aCC boutons in control animals **(G,H)**, but not in Dimm **(I,J)** and Dimm; dInR **(K,L)**. The position of the aCC neuron is indicated by arrows. An adjacent axon termination (on adjacent muscle) is strongly labeled in **(I,J)**.

It has been found that wingless signaling is critical for the establishment and plasticity of synapses at the NMJ (Packard et al., 2002; Collins and DiAntonio, 2007; Korkut et al., 2009). Wingless signaling provides an interaction between the pre- and post-synaptic components of the NMJ and the ligand wingless (Wg) released in exosome vesicles acts on the receptor frizzled-2 (dFz2) in the pre- and post-synaptic muscle membrane (Packard et al., 2002; Koles et al., 2012). Since *Dimm* expression targeted to aCC neurons disrupts expression of post-synaptic markers such as Dlg and GluRIIA and B in M1 and induces formation of filopodia-like processes, we tested the effects of ectopic *Dimm* on Wg and dFz2 immunolabeling. We found that both markers are almost completely eliminated in association with aCC neurons in M1 (**Figures 6C, 10**). The combined *Dimm; dInR* expression produces a complete loss of labeling (**Figures 10E,F,K,L**).

Next we sought a link between Dimm and wingless signaling and noted that some direct targets of Dimm may be involved in exosome release (see Hadzic et al., 2015). The exosome-dependent release of Wg requires the transmembrane protein Evenness Interrupted (Evi) (Koles et al., 2012). Two further proteins involved in Evi-exosome release, Rab11 and Ykt-6 (Gross et al., 2012; Koles et al., 2012), were shown to be direct targets of Dimm (Hadzic et al., 2015). Thus we tested effects of *Dimm* expression on Evi distribution in the NMJ. The pre- and post-synaptic immunolabeling with Evi N-terminal (Evi-Nex) antiserum was strongly diminished after *Dimm* and *Dimm; dInR* expression (**Figure 6C** and Supplementary Figures 6A–F), suggesting that exosome signaling may be affected.

### Mis-expression of Dimm in Motor Neurons Upregulates Expression of Known Dimm Targets

**FIGURE 11:**
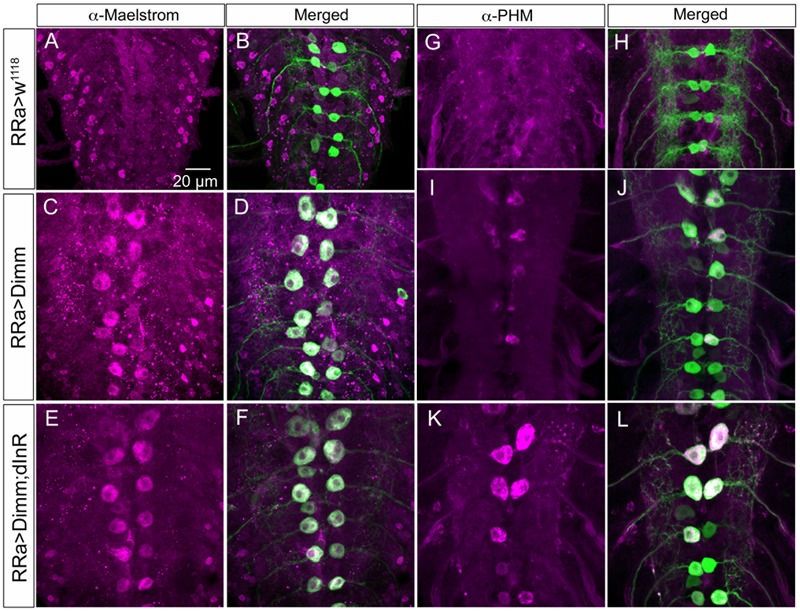
The Dimm targets maelstrom and PHM increase in aCC neurons after Dimm expression. **(A–F)** The aCC neurons express maelstrom immunolabeling after ectopic expression of Dimm and Dimm+dInR **(C–F)**, but not in controls **(A,B)**. Scale bar in **(A)** is also for **(B–L)**. **(G–L)** Also PHM immunolabeling is triggered by Dimm expression and accentuated by co-expression of dInR **(K,L)**.

**FIGURE 12:**
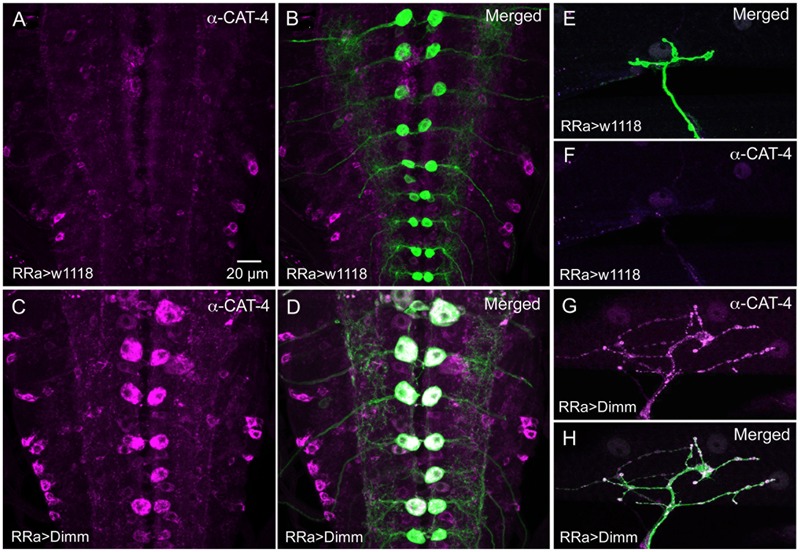
The Dimm target CAT-4 increases in aCC neurons after Dimm expression. **(A,B)** In controls CAT-4 immunolabeling is not detectable in the aCC neurons. Scale bar in **(A)** is also for **(B–H)**. **(C,D)** Ectopic Dimm expression in aCC neuron induces strong CAT-4 immunoreactivity in these cells. **(E–H)** Also in the axon terminations of aCC neurons CAT-4 immunoreactivity can be detected after Dimm expression. Data for effects of Dimm+dInR expression are shown in **Figure 6**.

We next asked what the immediate effects of *Dimm*-misexpression are on Dimm negative motor neurons. It has been shown that overexpression of *Dimm* leads to upregulation of a number of direct transcriptional targets (Park et al., 2011, 2014; Hadzic et al., 2015). In **Table 1** [data mined from (Hadzic et al., 2015)] we show Dimm targets with specific relevance for synaptic structure and morphology of the NMJ, others are related to secretory capacity. Expression levels of four Dimm target genes were tested here: CSP (Bronk et al., 2005), peptidylglycine-α-hydroxylating mono-oxygenase (PHM) (Jiang et al., 2000), maelstrom (Pek et al., 2009) and the vesicular transporter CAT-4 (Park et al., 2011). Membrane-associated CSP immunolabeling can be seen in aCC terminations of control larvae, but its distribution after *Dimm* and *Dimm+dInR* expression appears more diffusely cytoplasmic (Supplementary Figures 6G–P). The other three proteins are not expressed in detectable amounts in Dimm-negative neurons such as the aCC motor neurons (**Figures 6C, 11A,B,G,H, 12A–F**). Mis-expression of *dimm* and *Dimm+dInR* in aCC motor neurons, however, induces a strong immunolabeling with antisera to all three proteins (**Figures 6C, 11, 12**). PHM and CAT-4 immunolabeling can be detected both in the cell bodies and in the axon terminations of aCC neurons (**Figures 12E–H**), whereas maelstrom is only detectable in the aCC cell bodies (**Figures 11A–F**). Thus, Dimm triggers transcription of three of its known targets in the Dimm-negative aCC motor neurons and affects the distribution of CSP.

**Table 1:** Dimm target genes that affect growth of NMJ and synaptic structures.

Genes	Overexpression	RNAi or mutation	Reference
ACC (Acetyl-CoAcarboxylase)	–	Synaptic undergrowth	Valakh et al., 2012
Adf1 (Adh transcription factor 1)	Increased bouton number	Reduced bouton number	DeZazzo et al., 2000
ash2 (absent, small, or homeotic disks 2)	–	Pathfinding defect, reduced/abnormal synapses	Kraut et al., 2001
CG11486 (polyA-nuclease related protein)	Pathfinding defect, excess/ectopic synapses	–	Kraut et al., 2001
Chic (Chickadee)	–	Filopodia length reduced, axon extension reduced	Goncalves-Pimentel et al., 2011
Csp (Cystein string protein)	–	Reduced bouton number, string length, fewer branches, terminal length	Bronk et al., 2005; Dawson-Scully et al., 2007
Got2 (Glutamate oxaloacetate transaminase 2)	–	Reduced glutamate level, increased post-synaptic receptor area and more GluRs	Featherstone et al., 2002
Gs2 (Glutamine synthetase)	–	Fewer post-synaptic GluRs	Featherstone et al., 2002
Mbl (Muscleblind)	–	Synaptic undergrowth, synaptic retraction and axonal transport defect	Valakh et al., 2012
Pdk (Pyruvate dehydrogenase kinase)	Excess synapses, ectopic branches and axonal pathfinding errors	–	Kraut et al., 2001
sigA (Sluggish A)	–	Synaptic apposition, retraction and axonal transport defects	Valakh et al., 2012
Spi (Spitz)	–	Pathfinding defects, abnormal/reduced synapses	Kraut et al., 2001
Sta (Stubarista)	–	Synaptic apposition defect	Valakh et al., 2012
Vha55 (Vacuolar H^+^-ATPase 55 kD subunit)	–	Reduced Evi exosome release	Koles et al., 2012

*These genes were mined from a larger set in Hadzic et al. (2015).*

### Knockdown of Vesicular Glutamate Transporter Is Not Sufficient for Altering the Motor Neuron Phenotype

Since mis-expression of *Dimm* in motor neurons induced a down regulation of the glutamatergic phenotype, we asked whether knockdown of the vGluT is sufficient to trigger changes in NMJ morphology and expression of synaptic proteins. In RRa-Gal4 > UAS-vGluT-RNAi larvae we revealed a strong decrease in vGluT immunolabeling in the NMJ of the aCC neurons (Supplementary Figures 7A–F). However, we detected no obvious effect on neuron morphology or the expression of synaptic markers such as DLG (Supplementary Figures 7C,F) and GluRIIA (Supplementary Figures 7R–U). We also used the D42-Gal4 since it appears to be a stronger driver and obtained similar results (Supplementary Figures 7G–L). The D42-Gal4 was also used for knockdown of vGluT to monitor the effect on the cell bodies. No change was seen in cell body size, but the vGluT-immunolabeling was reduced (Supplementary Figures 7M–Q). In summary, our findings suggest that diminishing vGluT expression in aCC neurons (and RP2 neurons) is not sufficient to alter the motor neuron phenotype toward that obtained with *Dimm* mis-expression.

## Discussion

We have shown here that glutamatergic motor neurons can be altered toward a neuroendocrine phenotype by mis-expression of the transcription factor *Dimm*. This effect of *Dimm*-misexpression is enhanced by co-expression of the *dInR*. Thus, we validate and further develop the previously established notion that *Dimm* organizes a secretory phenotype in neurons, characteristic of neuroendocrine cells that store and secrete large amounts of neuropeptides or peptide hormones (Hewes et al., 2003; Park et al., 2008, 2011). By choosing glutamatergic motor neurons as a model we were able to use a wide set of morphological and molecular markers to monitor developmental changes, and also to build on a wealth of existing knowledge on NMJ and synaptic development in *Drosophila* (see Collins and DiAntonio, 2007; Menon et al., 2013).

The most obvious morphological alterations of motor neurons due to *Dimm* and *Dimm+dInR* expression was increased size of cell bodies, strongly reduced dendrites and increased branching of axon terminations with enlarged synaptic boutons at the NMJ. These transformed features taken together result in a morphology similar to that of efferent neuroendocrine cells rather than typical motor neurons. The one exception is the increased bouton size seen after Dimm expression; peptidergic neurons usually have much smaller boutons (Budnik, 1996; Keshishian et al., 1996). At the molecular level we noted a loss of several glutamatergic proteins (vGluT, GluRIIA, and GluRIIB) and as well as pre- and post-synaptic proteins (Brp, synapsin, synaptotagmin, and Dlg), all of which are commonly absent in association with efferent neuroendocrine cells. Interestingly, we also recorded filopodia-like extensions from the enlarged axon terminations, suggesting a change in interactions between motor neuron and target muscle. Such interactions involve wingless signaling, which also affects bouton size and number, as well as dGluRIIA receptor expression (Packard et al., 2002; Miech et al., 2008). In support of altered *trans*-synaptic signaling we indeed found that wingless/frizzled immunolabeling was strongly reduced in the *Dimm* mis-expressing axon terminations and at post-synaptic sites. Furthermore, expression of an exosome-associated protein, Evi, that is important for Wg exocytosis (Koles et al., 2012; Korkut et al., 2013) was diminished, and thus exosome secretion likely to be affected in aCC neurons after ectopic Dimm expression. It has been shown that the proteins Rab11 and Ykt-6, involved in Evi-exosome release at the NMJs (Gross et al., 2012; Koles et al., 2012) are Dimm targets (Hadzic et al., 2015). Rab11 is required for Evi-vesicle release at the NMJ and is localized at synaptic boutons and Wg is present in and secreted with Evi-exosome-like vesicles (Gross et al., 2012; Koles et al., 2012). The other component Ykt6, which is a prenylated ER-Golgi SNARE in mammals, is also required for exosome-dependent Wg secretion (Gross et al., 2012). Overexpression of Dimm in aCC motor neurons possibly interferes with the Ykt-6/Rab11 regulated exosome-dependent Wg secretion, and result in the disruption of wingless signaling at the terminals and thus a defect in synaptic formation and homeostasis.

In general, our findings on the effects of Dimm on motor neurons are similar to a previous investigation of effects of this transcription factor on *Drosophila* photoreceptors of the compound eye (Hamanaka et al., 2010). However, our study system provides access to further components of the targeted neuron, such as dendrite structure, and varicose, branching axon terminations with well-defined pre and post-synaptic proteins. The photoreceptor study demonstrated that *Dimm*-misexpression induces a loss of histamine expression in these receptors, as well as morphologically altered synaptic vesicles, loss of presynaptic T-bars, and enlarged and varicose axon terminations (Hamanaka et al., 2010). In comparison, we find a reduced expression of glutamatergic markers, loss of Brp expression, which represents active zone protein associated with the T-Bar (Kittel et al., 2006), and enlarged axon terminations and boutons. In addition, our study provides further data that suggest that post-synaptic alterations are also triggered by *Dimm* expression in motor neurons. Thus, we detected diminished SSR in the muscle fiber, as monitored by DLG immunolabeling, and reduction of glutamate receptor expression. In this context, we also obtained data suggesting a change in the reciprocal interactions between axon terminations and muscle cells that leads to filopodial outgrowth and expanded axon branching at the NMJ, probably linked to reduced or altered wingless signaling.

We attempted two other genetic manipulations of the motor neurons, but failed to induce a significant phenotype: we tried to mis-express three different neuropeptides, ion transport peptide, proctolin and short neuropeptide F (data not shown), and knocked down vGluT in motor neurons. It was actually shown earlier that vGluT mutant larvae display only subtle differences in bouton morphology in *Drosophila* motor neurons (Choi et al., 2014). Also in glutamic acid decarboxylase mutants that are unable to catalyze the formation of GABA from glutamate, the motor neuron axon terminations appear unaltered morphologically (Featherstone et al., 2000). Our lack of phenotypic changes after mis-expressing neuropeptides in motor neurons are not surprising, since it was shown earlier that ectopically expressing neuropeptide in photoreceptors had no effect unless *Dimm* was co-expressed (Hamanaka et al., 2010).

The effects of *Dimm* mis-expression are likely to start in the embryo, but seem to occur after axon outgrowth and target recognition, since the aCC neurons find M1 and form primary branch points in the correct position at the appropriate time. The aCC motor neuron axons reach muscle fibers of M1 in stage 16 (at 13 h) embryos and growth cones are seen at stage 16 and early stage 17, followed by bouton formation in second half of stage 17 (Yoshihara et al., 1997). Thus, the *Dimm* effects may commence at the time of establishing the neurotransmitter and presynaptic phenotypes, which occur between 14 and 16 h of embryonic development (Broadie and Bate, 1993; Keshishian et al., 1996; Yoshihara et al., 1997). At this time also the wingless signaling across the prospective synaptic cleft plays a critical role in formation of the functional synapse (Packard et al., 2002). Also the *trans*-synaptic changes seen after Dimm expression in aCC neurons (diminished Dlg, GluRIIA and B) and the altered wingless signaling may be arising at this stage. This could probably explain the filopodia-like protrusions seen in the axon terminations. The lack of a stable contact point seems to induce over-growth of the axon terminations, accompanied by filopodia-like protrusions, characteristic of growth cones normally seen in 13–16 h embryos (Yoshihara et al., 1997).

We found that some of the known transcriptional targets of *Dimm* were strongly up regulated after *Dimm* mis-expression: the synapse protein CSP, the peptide α-amidating enzyme PHM, the putative cationic amino acid transporter CAT-4 and maelstrom, a nuclear protein know to regulate micro-RNA (miR) (Zinsmaier et al., 1990; Hewes et al., 2006; Pek et al., 2009; Park et al., 2011). The first three of these are associated with the peptidergic secretory pathway and storage vesicles, whereas maelstrom is involved in germ line stem cell differentiation, and a role in neuronal development is not yet known (Pek et al., 2009; Park et al., 2011). It has been shown that knockdown of CSP in motor neurons impairs neurotransmitter release but also diminishes axon termination growth (Bronk et al., 2005; Dawson-Scully et al., 2007). Thus, there may be a link between Dimm-induced changes in CSP and growth of the axon terminations in our experiments. It was shown previously that maelstrom is highly enriched in Dimm-positive clock neurons (sLN_v_s), but its function was not revealed (Kula-Eversole et al., 2010). **Table 1** shows several transcriptional targets of Dimm (Hadzic et al., 2015) that have been shown to affect the differentiation of axon terminations and synaptic structure of *Drosophila* motor neurons. These include genes known to influence level of glutamate, glutamate receptor expression, number of axon branches, bouton number or size, synaptic growth, length of filopodia and axon pathfinding (references in **Table 1**). Hence, there are further candidates to test for roles in aCC motor neuron differentiation and effects of Dimm expression on synaptic development.

The central processes of the aCC neurons (and other motor neurons) display molecular and developmental features in common with dendrites of mammalian neurons (Sanchez-Soriano et al., 2005; Rolls, 2011). We noted a dramatic loss of dendritic branches of aCC neurons in the central neuropil after *Dimm* mis-expression. Thus, *Dimm* induces growth of cell body and axon terminations, but reduces dendrites. Scaling and growth of dendrite arborizations is regulated by factors like ecdysone receptors (Zwart et al., 2013), and Down syndrome cell adhesion molecule (Dscam1) (Hutchinson et al., 2014; Ryglewski et al., 2014). A few of the Dimm target genes (Hadzic et al., 2015) have been indicated in control of dendrite branching: *clockwork orange* (*cwo*), *Ef1alpha48D, mbf1, muscle blind* (*mbl*) and *Sin3A* (Parrish et al., 2006; Iyer et al., 2013; Olesnicky et al., 2014). These target genes, and others including wingless components (Valnegri et al., 2015), would also be of interest to investigate in relation to the Dimm-induced dendrite growth deficiency in aCC neurons.

In *Drosophila* as well as in mammals, several factors are known that act on growth of dendrites and axon terminations, and these portions of the neuron can grow independently of each other (Rolls, 2011; Wang et al., 2014; Valnegri et al., 2015). Effects on growth of dendrites, or other parts of neurons, have also been demonstrated after overexpression of phosphoinositide 3-kinase (PI3K) and tuberous sclerosis-2 (TSC2; Gigas), both downstream to tyrosine kinase signaling (Martin-Pena et al., 2006; Acebes and Morales, 2012; Luo et al., 2013). The PI3K induced growth can occur in absence of Dimm (Luo et al., 2013). However, expression of dInR has no effect on growth of neurons without co-expression of Dimm (Luo et al., 2013; Liu et al., 2016), suggesting that the PI3K mediated growth is induced by signaling other than insulin-like peptide. Interestingly, we could show here that ectopic expression of *dInR* combined with *Dimm* amplifies most phenotypes seen after *Dimm* expression alone. Thus, an interesting question for the future is to determine how *Dimm* and *dInR* cooperate to regulate expression of Dimm targets and establish an endocrine phenotype.

## Author Contributions

JL, YL, and DN: designed the research; JL and YL: performed the research; JL and YL: performed main data analysis; JL, YL, and DN: drafted the manuscript; DN: edited final version (the other authors read and approved the final version of the manuscript); DN: supervised the study and obtained funding.

## Conflict of Interest Statement

The authors declare that the research was conducted in the absence of any commercial or financial relationships that could be construed as a potential conflict of interest.

## Footnotes

1 http://rsb.info.nih.gov/ij/
