# Supplementary material for: Transcriptional Reorganization of Drosophila Motor Neurons and Their Muscular Junctions toward a Neuroendocrine Phenotype by the bHLH Protein Dimmed
Source: Front Mol Neurosci. 2017 Aug 14;10:260. doi: 10.3389/fnmol.2017.00260 (PMC5557793; doi:10.3389/fnmol.2017.00260)
Supplement: Supplementary file 1 [file Image_1.PDF]

Supplementary Material

Transcriptional reorganization of *Drosophila* motor neurons and their muscular junctions towards a neuroendocrine phenotype by the bHLH protein Dimmed

Jiangnan Luo, Yiting Liu, Dick R Nässel

*** Correspondence:** Dick R Nässel, Department of Zoology, Stockholm University, SE-10691 Stockholm, Sweden, e-mail: dnassel@zoologi.su.se

1 Supplementary Figures

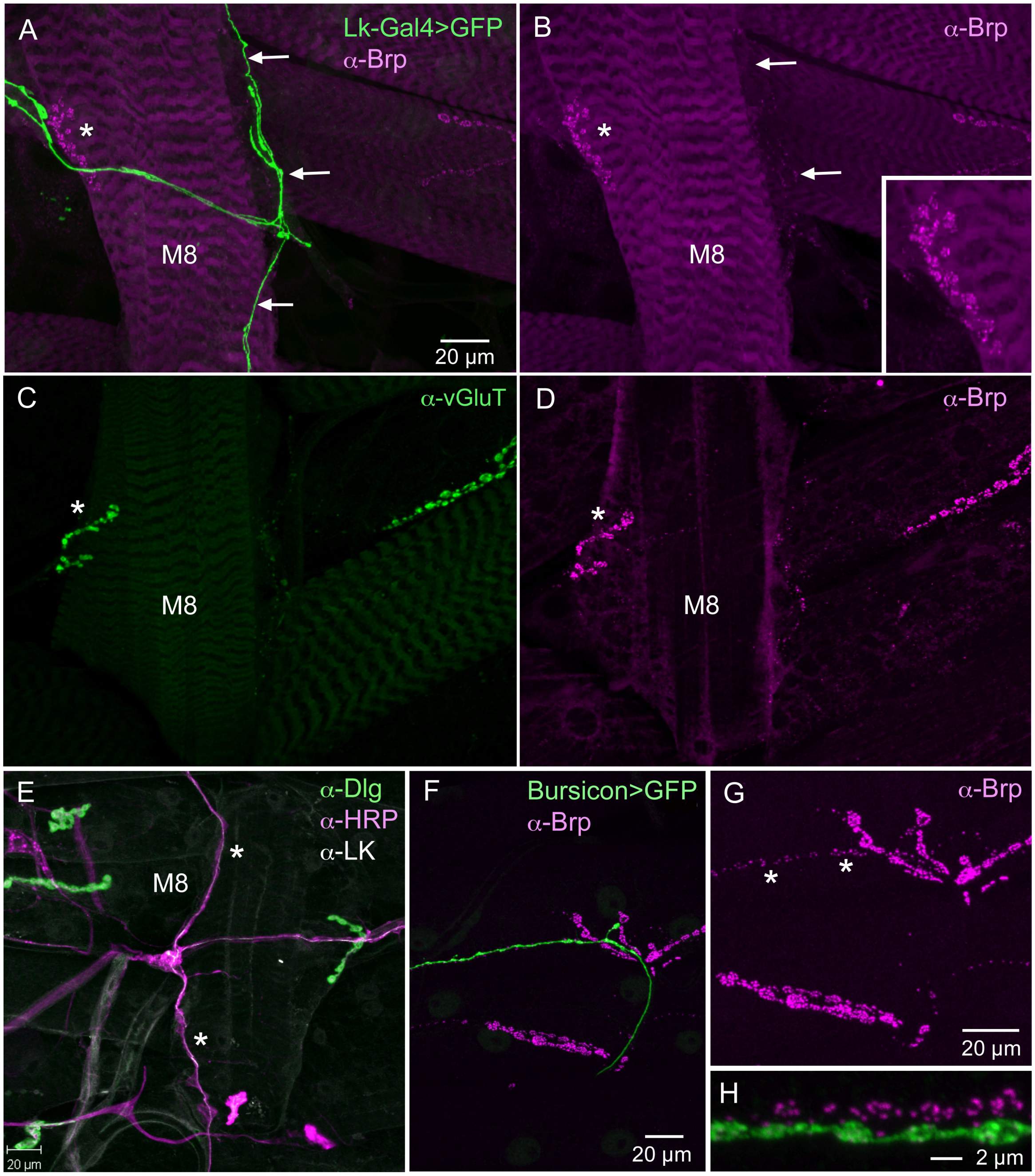

Suppl. Fig. 1. Axon terminations of peptidergic efferent neurons express very little bruchpilot (Brp) and are not associated with discs large. **A-B**. Immunolabeling with anti-Brp and expression of Lk-gal4 driven GFP. The arrows indicate the main axon termination associated with M1. Asterisks indicate Brp expression. **C-D**. Labeling with vGluT antiserum (C) and Brp (D) is mainly associated with leucokinin-negative axon terminations (away from M1). **E**. Triple labeling with antisera to HRP, discs large (Dlg) and leucokinin (LK). The main axon termination of LK neuron (asterisks) does not express Dlg. **F-H**. Axon termination on abdominal body wall muscle expressing the neuropeptide bursicon (Burs-Gal4>GFP) does not express Brp immunolableing. The punctate labeling see at asterisks is primarily outside the busricon expressing axon termination (see H for detail).

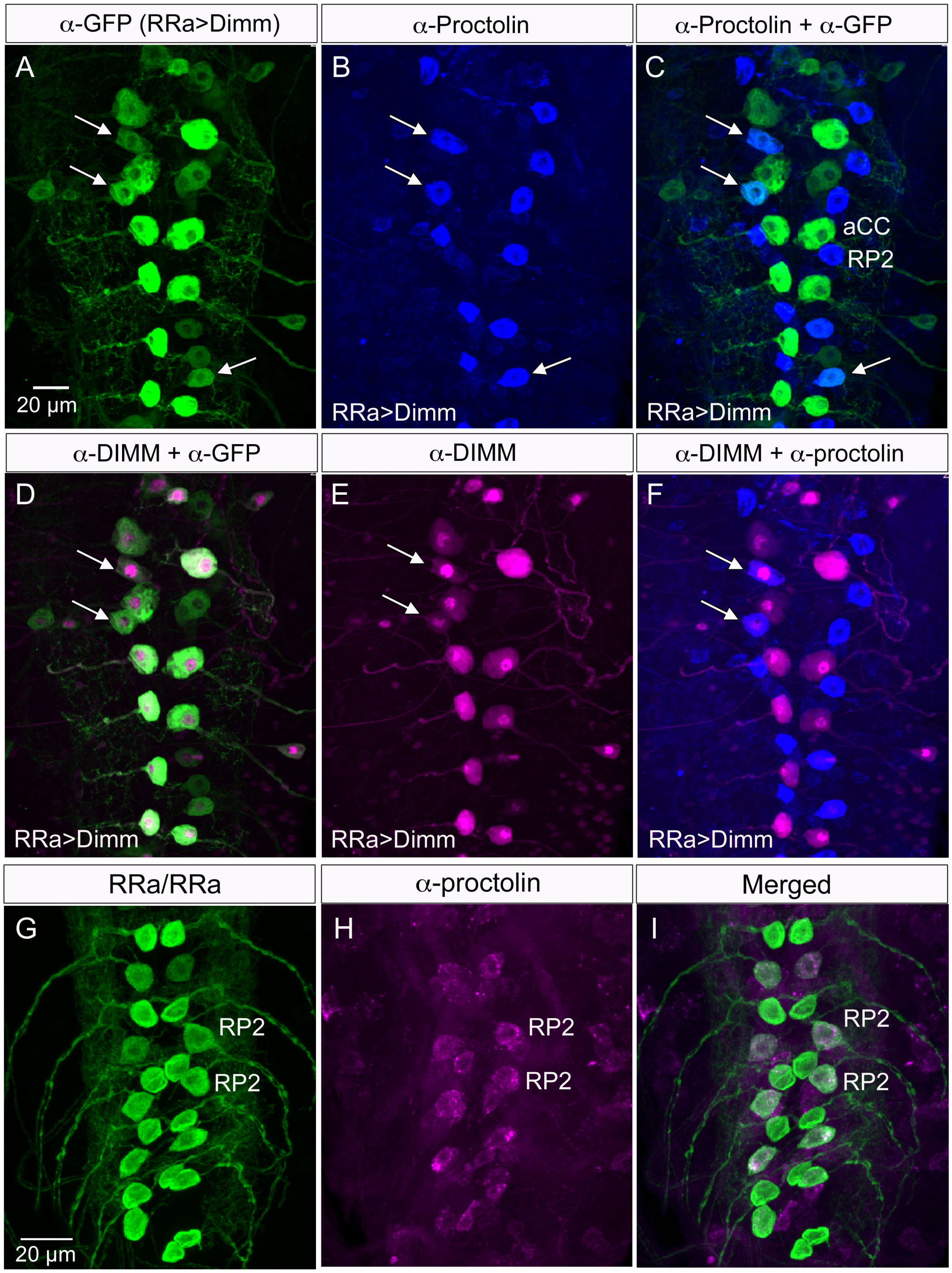

Suppl. Fig. 2. The aCC neurons are not proctolinergic even if ectopically expressing Dimm. **A-C**. In RRa>Dimm larvae there is no proctolin immunolabeling of aCC neurons. However the adjacent RP2 neurons express proctolin (as they do in wild type larvae (see G-H)). The neurons indicated by arrows are RP2 neurons that still express GFP in the RRa-Gal4 line at third instar larval stage. **D-F**. The aCC neurons express ectopic DIMM immunolabeling coinciding with GFP. The two RP2 neurons that continue to express GFP display DIMM labeling and proctolin (indicated by arrows). **G-I.** In homozygous RRa-Gal4 lines the RP2 neurons are visible with anti-GFP and label with antiserum to proctolin.

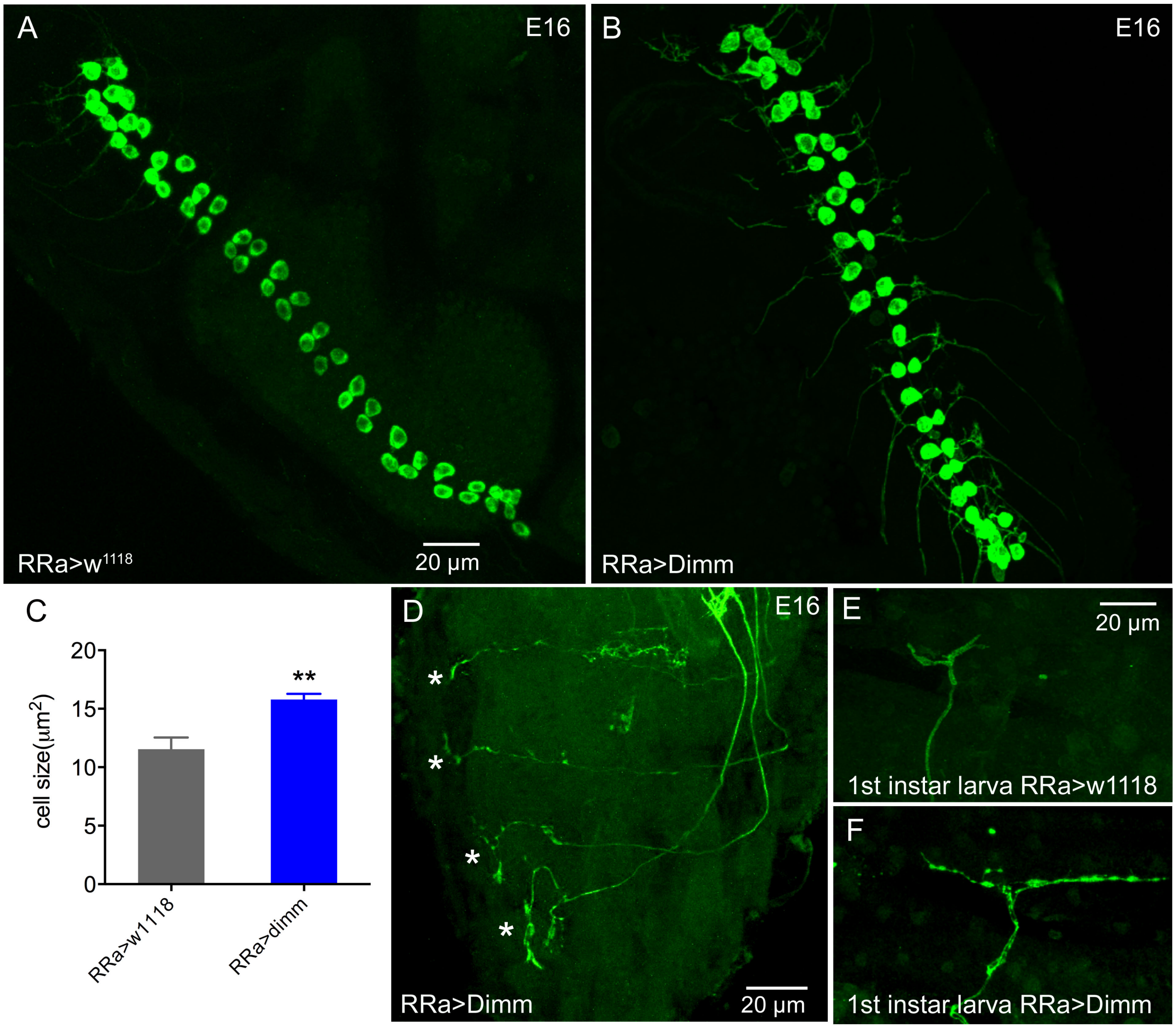

Suppl. Fig. 3. Developmental effects of ectopic Dimm expression. **A-B.** In stage 16 embryos (E16) ectopic Dimm expression increases the size of aCC and RP2 cell bodies. **C**. Quantification of increase in cell body size of aCC and RPs neurons. **D**. At stage E16 the axon terminations of the aCC/RP2 motor neurons have expanded on the target muscles. **E-F**. In first instar larvae ectopic Dimm expression increases axon terminations of aCC neurons.

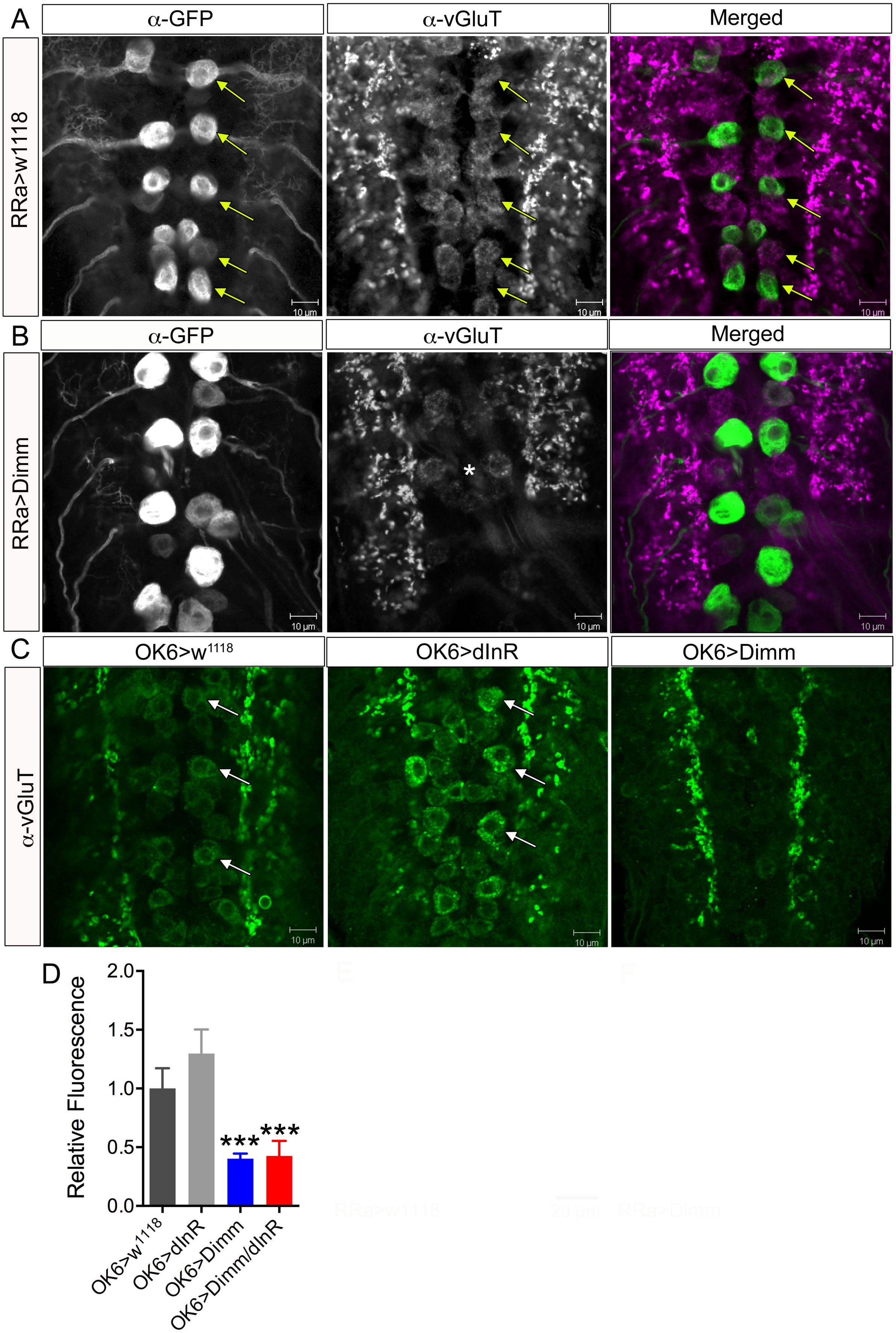

A
α-GFP
α-vGluT
Merged
RRa>w1118
10 µm
B
α-GFP
α-vGluT
Merged
RRa>Dimm
*
10 µm
C
OK6>w1118
OK6>dInR
OK6>Dimm
α-vGluT
10 µm
D
Relative Fluorescence
2.0
1.5
1.0
0.5
0.0
***
***
OK6>w1118
OK6>dInR
OK6>Dimm
OK6>Dimm/dInR

Suppl. Fig. 4. Ectopic Dimm expression diminishes vesicular glutamate transporter (vGluT) immunolabeling in motor neurons. **A-B**. Using the RRa-Gal4 driver for Dimm expression we show that vGluT immunolabeling diminishes compared to controls (RRa>w^{1118}). The cell bodies that do express vGluT immunolabeling are likely from RP2 neurons (note that they do not grow, in contrast to the GFP-labeled aCC neurons). **C**. Using a wider Gal4 driver, OK6, we show that targeting the insulin receptor (dInR) increases expression of vGluT immunolabeling, whereas Dimm strongly decreases in. **D**. Quantification of reduced vGluT-immunolabeling seen after driving Dimm and Dimm; dInR with the OK6 Gal4 driver. Data are presented as means ± S.E.M; with 7-9 larvae from three biological replicates (**p<0.01, ***p<0.001), as assessed by one way ANOVA test followed by Dunnett's multiple comparisons test).

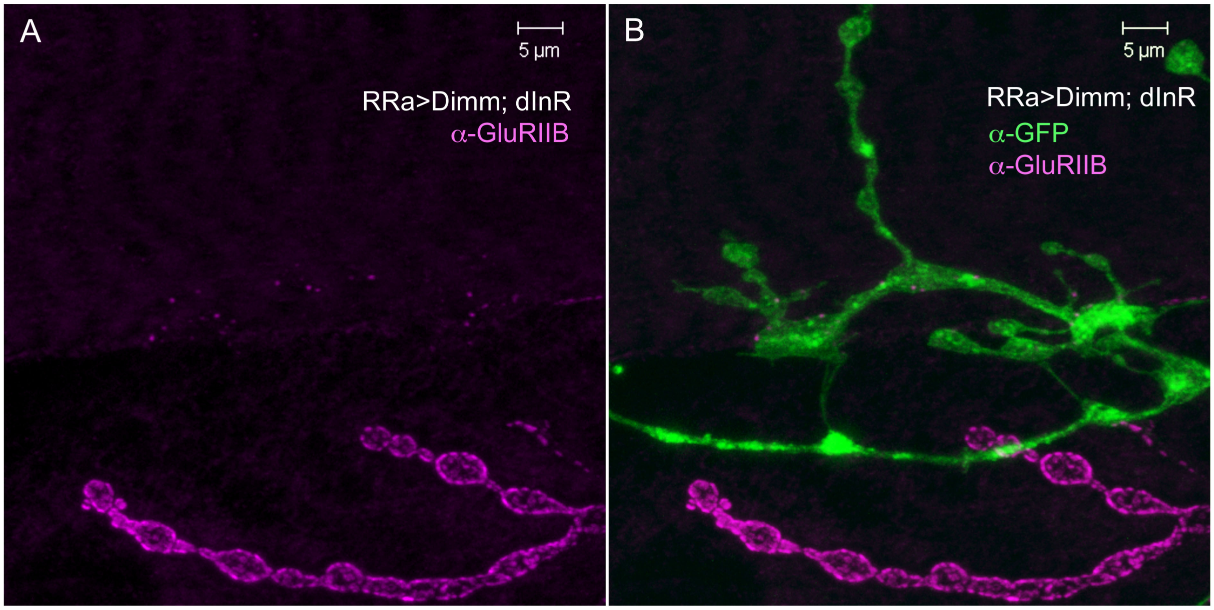

Suppl. Fig. 5. The glutamate receptor GluRIIB immunolabeling diminishes after targeting Dimm; dInR to aCC neurons. **A-B**. GluRIIB immunolabeling is nearly completely lost after Dimm was targeted to aCC neurons. An adjacent axon termination from another motor neuron is seen below with strong GluRIIB immunolabeling. (To accompany Fig. 9).

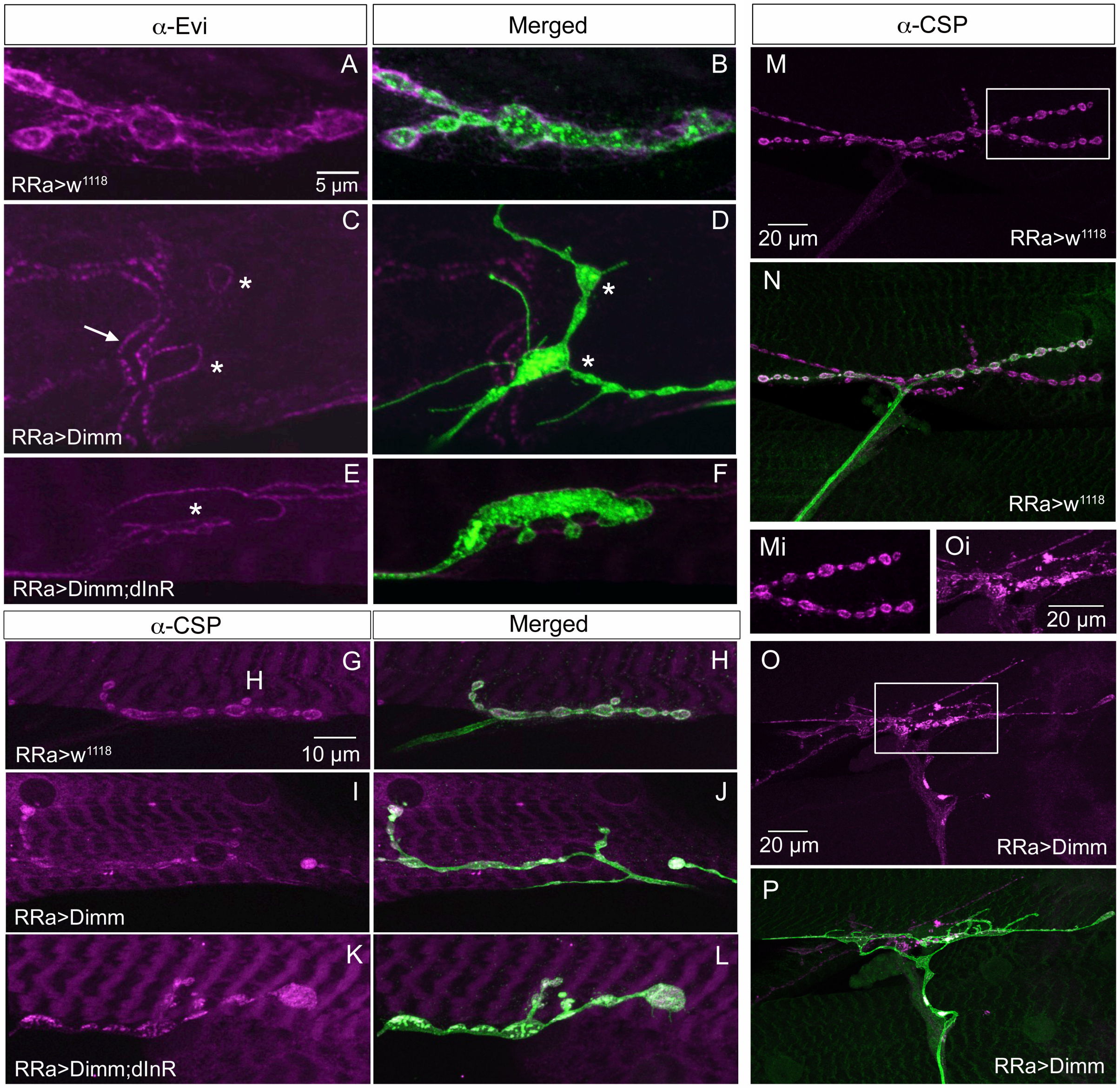

Suppl. Fig. 6. Eveness interrupted (Evi) and cysteine string protein (CSP) in axon terminations are affected by ectopic Dimm expression. **A-F**. Immunolabeling with antiserum to Evi is strong in and around boutons of control aCC neurons (A, B). After ectopic expression of Dimm (C, D) and Dimm;dInR (E, F) the aCC boutons loose most of the Evi immunolabeling; some residual labeling can be seen around the larger boutons (asterisks), and in adjacent axon terminations (arrow). **G-L**. The presynaptic cysteine string protein (CSP) is seen adjacent to membranes in aCC boutons of control animals (G, H). After expression of Dimm (I, J) and Dimm;dInR (K, L) the aCC boutons seem to accumulate more cytoplasmic CSP immunolabeling. **M-P**. Further images to show the redistribution of CSP after ectopic Dimm expression in aCC neuron terminations. The boxed areas in **M** and **O** are shown in higher magnification in Mi and Oi. Note the shift from membran distribution to diffuse cytoplasmic.

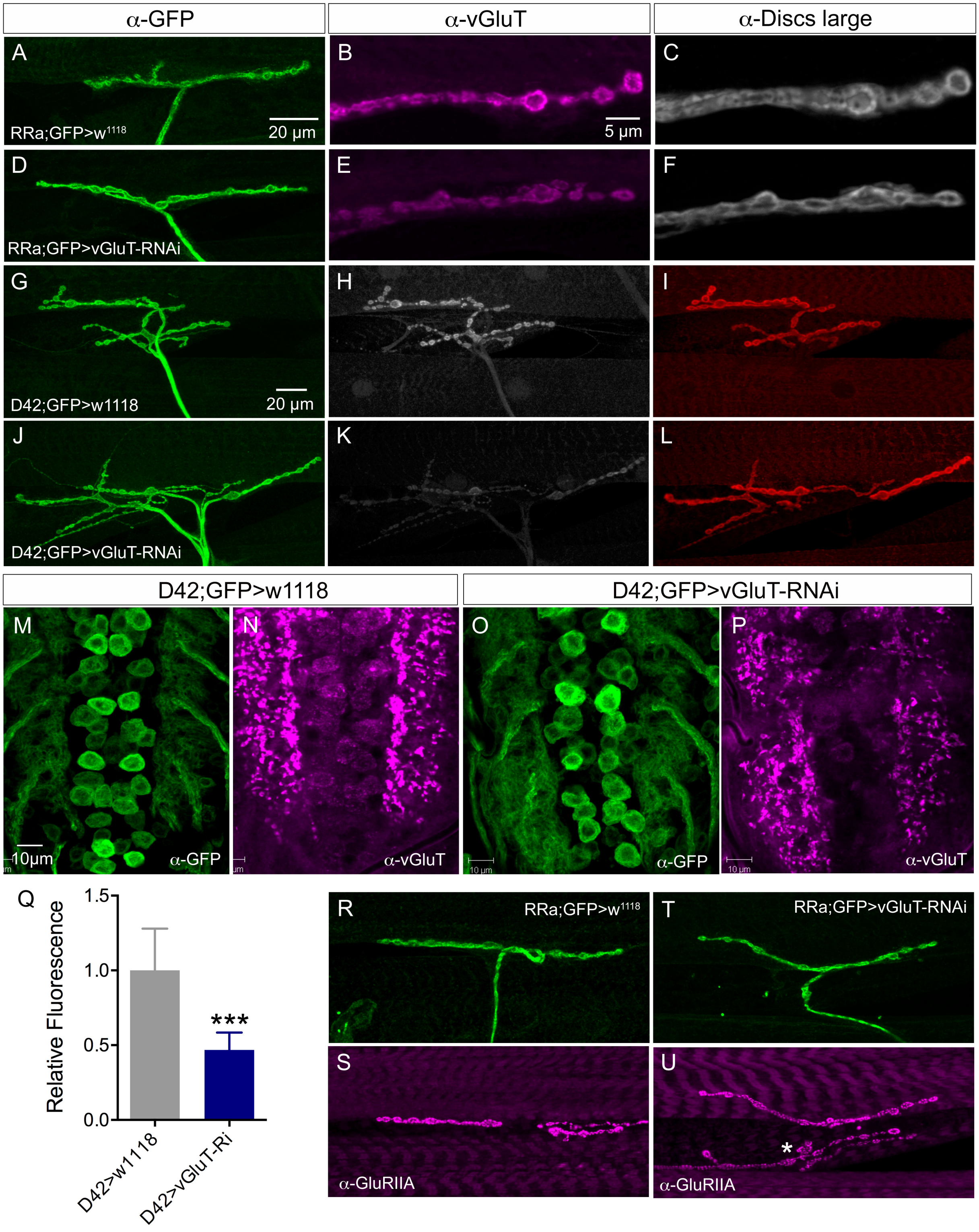

Suppl. Fig. 7. Knock down of vGluT does not affect the size of axon terminations or cell bodies of motor neurons. **A-F**. Using the RRa-Gal4 to drive vGluT-RNAi we note a decrease in vGluT immunolabeling, but no

effect on axon termination or bouton size. **G-L**. With a stronger and more widely expressing driver, D42, the vGluT immunolabeling is further diminished, but still no effect on axon termination size was noted. M-P. The D4 driven vGluT-RNAi diminishes vGluT-immunolabeling also in cell bodies of motor neurons, but this does not affect cell body size. **Q**. Quantification of reduced vGluT-immunolabeling in axon terminations seen after driving vGluT-RNAi with a strong motor neuron driver, D42-Gal4. Data are presented as means ± S.E.M; with 10 larvae from three biological replicates (***p<0.001), as assessed by unpaired student's test). **R-U.** Driving vGluT-RNAi in aCC neurons does not affect expression of the ionotropic glutamate receptor subunit GluRIIA.
